# Antibiotic Resistance as a Functional Characteristic of Urban Dust Particles’ Microbial Communities

**DOI:** 10.3390/biology13121022

**Published:** 2024-12-06

**Authors:** Anna A. Vetrova, Anastasia A. Ivanova, Kirill V. Petrikov, Olga Gavrichkova, Maria V. Korneykova, Olesya I. Sazonova

**Affiliations:** 1 Federal Research Center “Pushchino Scientific Center for Biological Research of the Russian Academy of Sciences”, 142290 Pushchino, Russia; mrs.ivanova.a.a@gmail.com (A.A.I.); bioscience.kp@gmail.com (K.V.P.); sazonova_oi@rambler.ru (O.I.S.); 2 Research Institute on Terrestrial Ecosystems, National Research Council, 05010 Porano, Italy; olga.gavrichkova@cnr.it; 3 Agrarian and Technological Institute, Peoples’ Friendship University of Russia (RUDN University), 117198 Moscow, Russia; korneykova.maria@mail.ru; 4 Institute of North Industrial Ecology Problems Subdivision of the Federal Research Center “Kola Science Centre of Russian Academy of Science”, 184209 Apatity, Russia

**Keywords:** metabarcoding, 16S rRNA, antibiotic resistance, microbiome, PICRUSt, FAPROTAX

## Abstract

Urban dust samples were collected in Moscow (Russia) in June 2021. The samples were collected in three functional zones of Moscow (traffic, residential, and recreational) and included air microparticles, leaf dust, and paved dust. Data on the taxonomic composition of bacterial communities were obtained for dust samples, and their functional characteristics were predicted using PICRUSt2 2.5.0 and FAPROTAX 1.8.0 software. The culturable part of the bacterial community was examined for the presence of antibiotic-resistant strains with respect to β-lactams, tetracyclines, amphenicols, and aminoglycosides. The presence of bacteria resistant to ceftazidime, cefepime, and tetracycline was detected in all dust samples. The presence of bacteria resistant to meropenem and amikacin was only observed in the dust collected from leaves in the residential and traffic zones. The overall abundance of cultured antibiotic-resistant bacteria from the total heterotrophs ranged from 0.03% to 1.88%, with the highest percentage observed in dust from the residential zone. Notably, strains resistant to all antibiotics tested were observed in the leaf dust bacterial community.

## Simple Summary

Air pollution has been identified as a contributing factor to increasing antibiotic-resistant bacteria in the microbiome of dust particles. The aim of the study was to structurally and functionally characterise the microbial communities of dust particles in urban ecosystems of Moscow and to compare the prevalence of predicted resistome modules and culturable antibiotic-resistant bacteria in the microbiomes. The present study revealed a higher prevalence of antibiotic-resistant bacteria in the microbiomes of dust particles sampled in the most polluted functional zones of Moscow. Furthermore, antibiotic resistance was a more diverse functional characteristic of the microbial communities of airborne particles.

## 1. Introduction

More than half of the world’s population lives in urban areas. They increasingly live in densely populated areas [1]. This increases the possibility of the transfer of bacteria (including opportunistic and pathogenic bacteria), antibiotic-resistant bacteria, and antibiotic resistance genes into urban ecosystems [2]. Urban particulate matter is one way of transporting these elements [3]. These particles can be deposited on various surfaces and travel considerable distances on air currents [4]. This leads to the exchange of microorganisms between microbial communities of different urban areas and biotopes [5]. It should be noted that because dust particles penetrate deeper into the body through the respiratory system and skin pores, the smaller the particles, the greater the negative impact on human health.

Antimicrobial resistance among healthcare-associated and nosocomial pathogens has increased worldwide in recent years [6]. The emergence of resistance is a natural biological response to the use of antimicrobial drugs. Antimicrobial drugs exert a selective pressure that favours the selection, survival, and proliferation of resistant strains of microorganisms.

Importantly, microorganisms carrying antibiotic resistance genes have been found in various environmental samples, including freshwater [7], soil, sediments [8], and various urban facilities such as wastewater treatment plants [9,10] and hospitals [11,12]. Recently, microbial communities from outdoor [13,14] and indoor dust [15] have also been found to contain genes associated with antibiotic resistance.

According to a study by Zhou et al. [16], deterioration of air quality increases antibiotic resistance. This is because antibiotic-resistant microorganisms are present in the biogenic fraction of particulate matter. Metals, biocides, pesticides, and other chemicals can stress microorganisms and facilitate the adaptation of members of microbial communities to changing environmental conditions. This can lead to a redistribution of microorganisms in terms of dominance and favour the development of resistant members of the community. One way of adapting is to increase the representation of antibiotic-resistant microorganisms in the community. Therefore, researchers have found that reducing air pollution can help reduce antibiotic resistance.

Rosas et al. [14] investigated the faecal contamination of settled dust from indoor and outdoor urban areas. The article measured and characterised dust samples for the presence of faecal coliforms using *Escherichia coli* virulence genes, adhesion properties, and antibiotic resistance as markers. The authors found that the number of strains resistant to at least one antibiotic was two times higher in dust samples collected in open urban areas than in closed urban areas. Multidrug resistance to different antibiotics was more common in the majority of these strains. The data obtained by Rosas et al. [14] highlighted the need to study airborne microbial communities to assess their functional activity in relation to antibiotic resistance.

Therefore, there has been an increase in the study of the resistome in recent years [17,18]. The resistome is the set of all genes that confer resistance to antibiotics and includes those genes that are present in both pathogenic and non-pathogenic microorganisms. The resistome includes genes that are normally found in nature [19,20], genes that arise from selective exposure to antibiotics, novel (man-made) resistance genes that arise from pre-resistance genes, and cryptic genes that are already present but not expressed [19]. As part of mobile genetic elements, all these structures are amplified and propagated within the microbial population by mutation and horizontal gene transfer [21].

The present work investigated the microbiomes of airborne dust particles, plant phylloplane, and paving dust, including with respect to predicted antibiotic resistance functions. Dust particles were sampled in three zones: recreational, residential, and traffic (traffic zone). This is due to the concept of the functional zoning of cities, which includes an increasing anthropogenic impact on human life and health. Chemical pollutants in the air and their effects on human health have been widely studied [22]. However, the taxonomic analysis of the microbial populations in the air, especially the antibiotic-resistant strains, is still underestimated. Most studies of urban airborne antibiotic resistance genes have focused on sources such as hospitals [23], wastewater treatment plants [24], or the effects of the presence or absence of atmospheric smog [25]. There is still a lack of understanding of the spatial and urban distribution of antimicrobial resistance within dust particle metagenomes in different functional zones of the city, including their circulation between environmental biotopes. A more detailed profiling of dust particle metagenomes would improve our understanding of potential health risks due to routine exposure of the general population, considering the analysis of their functional activity in relation to antibiotic resistance at the urban scale. Thus, the aims of this work were to describe the structural and functional characteristics of the microbial communities of dust particles in the urban environment of Moscow and to compare the distribution of predicted resistome modules and cultured antibiotic-resistant bacteria in the microbiome.

## 2. Materials and Methods

### 2.1. Site Description

This study was conducted in Moscow, the largest city in Russia, with an area of 2600 km^2^. During the study in June 2021, dust particles (PM10 from air; PM100 from leaf surfaces and paved roads) were sampled in three districts (zones) of the city. The selected areas differ in the degree of anthropogenic influence. The area with the lowest level of pollution was the park (recreational) zone, including the historical park named after K.A. Timiryazev and the dendrological garden named after R.I. Schroeder (55.833000 N, 37.549794 E). The site with the highest level of anthropogenic impact (55.738328 N, 37.620061 E) was located near the Garden Ring. This is one of the main motorways in the central part of the metropolis (traffic zone). The site with an intermediate level of anthropogenic pollution (55.651983 N, 37.499363 E) was located on the campus of the Peoples’ Friendship University of Russia, named after P. Lumumba (RUDN) (residential zone). It is far from the city’s main motorways. The difference in the level of anthropogenic pollution was already confirmed in Korneykova et al. [26]. Sampling was conducted at least one week after raining to exclude the influence of atmospheric precipitation.

### 2.2. Sampling

We studied three different urban zones: a recreational zone (conditionally clean/control zone), a residential zone, and a traffic zone (two zones with different levels of anthropogenic load). Particulate matter (PM) samples were collected in three functional zones from three different biotopes: air, paved, and the leaf surface of the woody plant *Betula pubescens* Ehrh. In total, nine samples were collected.

(1) PM10 polycarbonate filters. For the sampling of. PM10 from the air, special samplers with a flow rate of 10 L/min (SKC, Dorset, UK) were installed at a height of 3 m. These samplers contained an impactor that allowed the airborne PM to be fractionated so that only particles of 10 µm or less passed through. Polycarbonate filters with a diameter of 47 mm and a pore size of 0.8 µm, supplied by Sterlitech Corporation (Auburn, WA, USA), were used to collect particles in the range of 1 µm to 10 µm. To increase the amount of sample collected, PM10 was collected over 8 days, with filters changed every 48 h as recommended by Ferguson et al. [27]. Each polycarbonate filter was weighed before and after PM10 collection. The difference in mass was used to determine the amount of dust deposited on the filter.

Thus, eight filters with air PM10 were collected from each urban area. Four filters pooled together were used for metabarcoding, and the other four filters (also pooled together) were used to analyse culturable heterotrophic bacteria (including antibiotic-resistant bacteria).

(2) Paved road surface. Dust from paved surfaces (asphalt) was collected according to the method described by Pollegioni et al. [28];
(3) Leaf surface. Leaves of *Betula pubescens* Ehrh. were collected to study the bacterial communities of dust particles deposited on the surface of tree leaf plates. Dust from the leaves of *Betula pubescens* Ehrh. was collected according to the method described by Pollegioni et al. [28].

Dust particles were washed from the leaves under laboratory conditions, as described in detail in Section 2.3.

All procedures were carried out under aseptic conditions. For metabarcoding, samples were frozen and stored at −20 °C until further processing. To analyse the culturable part of the bacterial community (including antibiotic-resistant bacteria), samples of dust particles were used for the experiment immediately after the end of sampling.

### 2.3. Dust Removal from Leaf Surface

(1) The leaves of *Betula pubescens* Ehrh. (approximately 1000–1100 cm^2^) were placed in 300 mL of physiological solution and stirred vigorously for 15 min at 28 °C, and 200 rpm for the metabarcoding of bacterial dust communities was deposited on the phylloplane. Nylon filters with a pore size of 100 μm were used for the removal of large contaminants (e.g., small leaf plate particles). To extract the dust fraction from the resulting filtrate, sterile vacuum filter funnels with a pore size of 0.22 μm (Thermo Scientific, Waltham, MA, USA) were used. Filters with dust particles were carefully cut out and stored at −20 °C until further processing. ImageJ 1.54g software was used to count leaf surface area;
(2) In order to analyse the culturable part of the bacterial communities of phylloplane dust, leaves with a total surface area of about 550–650 cm^2^ were placed in 100 mL of physiological solution, and then, the procedure described in the previous paragraph (1) was followed;
(3) In order to determine the amount of dust deposited on the surface of the leaf plates used for the analyses, the total leaf area of approximately 280–350 cm^2^ was placed in 25 mL of deionised water and stirred vigorously for 15 min at 28 °C and 200 rpm. The suspension with dust was transferred to pre-weighed glass vials after removal of coarse impurities and determination of the volume of drained liquid. The vials were placed in a thermostat at 65 °C until the liquid evaporated completely. The vials with dust were then weighed. As a result, the mass of dust deposited on 1 cm^2^ of leaf plate was determined.

### 2.4. DNA Extraction and 16S rRNA Gene Metabarcoding 

Total genomic DNA samples were isolated from the collected dust using the DNeasy PowerSoil Kit (Qiagen, Hilden, Germany). The manufacturer’s recommendations were followed for all procedures. The concentration of the isolated DNA and its quality were assessed using the NanoDrop Opec spectrophotometer (Thermo Scientific, Waltham, MA, USA). The V3–V4 hypervariable regions of the bacterial 16S rRNA gene were amplified using 341F (5′-CCTACGGGGGNGGCWGCAG-3′) and 805R (5′-GACTACHVGGTATCTAATCC-3′) primers [29]. Sequentia Biotech SL (Barcelona, Spain) performed the polymerase chain reaction, library preparation, and subsequent sequencing on the Illumina MiSeq platform (paired end, 2 × 300 cycles).

### 2.5. Bioinformatic Analysis

The bioinformatic processing of the data was performed using the R v4.2.1 programming language (R Development Core Team 2022, Vienna, Austria) and the associated packages. In brief, the DADA2 package was used to generate amplicon sequence variants (ASVs) from paired-end demultiplexed reads [30]. First, primers were removed. After checking the quality of the sequences, the forward and reverse reads were trimmed to 280 bp and 260 bp, respectively. The forward and reverse reads were then combined, and chimeric sequences were removed. The SILVA database v. 138.1 [31] was used for the taxonomic classification of the ASVs obtained. ASVs that could not be classified at the kingdom level and those belonging to chloroplasts or mitochondria were removed from further analyses of the bacterial communities of urban dust in Moscow.

### 2.6. Determination of the Number of Culturable Bacteria

To determine the total number of culturable bacteria (CFU/mg of sample) in urban dust samples, we used the approach described by Sazonova et al. [32]. PCA (Condalab, Madrid, Spain) was used as the nutrient medium.

The preparation of urban dust samples for plating on nutrient media was as follows:

(1) PM10 air. For each functional zone, four air filters containing PM10 were pooled and placed in a test tube containing 4.5 mL of physiological solution (0.9% NaCl). The contents of the tube were then thoroughly mixed on a laboratory vortex to wash away dust particles. The resulting suspension was used to perform a series of dilutions and then plated on PCA medium (Condalab, Madrid, Spain);
(2) Dust from paved road. The mixed dust sample was used for analysis according to the method described by Sazonova et al. [32];
(3) Dust from leaf surface. Filters with dust particles deposited on their surface were used for analysis according to the method described by Sazonova et al. [32].

The dishes were incubated at 28 °C for 5–7 days. The number of culturable bacteria was determined by direct counting of colonies grown on agar.

### 2.7. Isolation and Identification of Culturable Antibiotic-Resistant Bacteria

The total number of culturable antibiotic-resistant bacteria (CFU/g sample) for each sample was determined by direct counting of colonies grown on PCA supplemented with one of the following antibiotics: meropenem (40 µg/mL), ceftazidime (40 µg/mL), cefepime (40 µg/mL), tetracycline (20 µg/mL), chloramphenicol (100 µg/mL), amikacin (30 µg/mL), kanamycin (100 µg/mL), streptomycin (100 µg/mL), or gentamicin (10 and 40 µg/mL). Antibiotic concentrations were selected based on the European committee for antibiotic susceptibility testing EUCAST database (http://www.eucast.org, accessed on 10 September 2024), as reported in the articles by Kosheleva et al. [9] and Izmalkova et al. [33]. Colonies grown on the individual antibiotics were tested for resistance to other antibiotics listed above by cross-replication. 

Genomic fingerprinting, according to the technique described by Sazonova et al. [32], was used for the differentiation of the isolates obtained. As a result of these manipulations, isolates from a dust sample with the same fingerprint, colony morphology, and antibiotic resistance were assigned to a strain or closely related strains of the same species (Table S1). It was these colonies of antibiotic-resistant bacteria with a different antibiotic spectrum or colony morphotype or fingerprint that were further identified based on 16S rRNA gene sequencing. To determine the nucleotide sequence of the 16S rRNA gene, genomic DNA samples were isolated from bacterial cells of individual strains using the Quick-DNA Fungal/Bacterial Miniprep Kit (Zymo Research, Irvine, CA, USA) according to the manufacturer’s protocol. Polymerase chain reaction (PCR) was then performed using two primers, 27F (5′-AGAGAGTTTGATCCTGGCTCAG-3′) and 1492R (5′-TACGGGGYTACCTTGTTGTTACGACTT-3′) [34]. PCR was performed on a GeneAmp PCR System 9700 cycler (Applied Biosystems, Waltham, MA, USA). The resulting PCR product was purified using a Cleanup S-Cap kit (Eurogen, Moscow, Russia).

To determine the nucleotide sequence of the obtained PCR-products, sequencing technology was applied using the ABI 3130 xl Analysis System manufactured by Applied Biosystems (USA). Sequencing was performed at Eurogen (Russia). The BLASTN resource available at http://blast.ncbi.nlm.nih.gov/ (accessed on 25 August 2024) was used to determine the identity of the nucleotide sequences.

### 2.8. Statistical Analysis

All statistical processing of the bacterial community results was performed using R v4.2.1. Briefly, using the rarefy function from the vegan v2.6-2 package, the ASVs tables were rarefied to the minimum library (34,705 reads) [35]. The following alpha diversity indices were calculated (phyloseq package): Chao1, Fisher, Simpson, Observed, and Shannon [36]. The package ggpubr v0.6.0 was used for visualisation, and significant differences were calculated using the package rstatix v0.7.2. A technique based on the calculation of the Bray–Curtis pairwise dissimilarity matrix was used to assess beta diversity. Analyses were performed at both the bacterial class and species level. At the class level, the similarities and differences in the composition of bacterial dust communities from different biotopes and functional zones were assessed by constructing a dendrogram. At the species level, beta diversity was assessed using principal coordinate analysis (PCoA) and multivariate non-metric scaling (NMDS). A non-parametric multivariate statistical permutation test (PERMANOVA) was performed, and the corresponding R-squared and *p*-values were calculated. The ggplot2 v3.5.1 package was used for data visualisation. 

To detect the presence of common ASVs in the studied communities of three biotopes of three functional zones, Venn diagrams were constructed [37].

We used the FAPROTAX 1.8.0 (Functional Annotation of Prokaryotic Taxa) database to identify differences in functional traits between the communities studied [38]. We used the online resource Morpheus [39] for visual processing of the results obtained; indicators were normalised using the min–max scaling principle, and correlations were calculated using Pearson’s linear correlation coefficients.

Using PICRUSt2 2.5.0 (Phylogenetic Investigation of Communities by Reconstruction of Unobserved States) software, we analysed the predicted functional profile of microbial communities for the possible presence of antimicrobial-resistance genes [40]. The resulting data on the representation of functional KO orthologs from the KEGG ORTHOLOGY database for each sample were used for further analysis. These results were analysed using the ggpicrust2 package (function ‘pathway_daa’, method ‘DESeq2’, Benjamini–Hochberg corrections for multiple comparisons). This analysis revealed differences in the representation of each KO orthologs between all variants of the sample combinations, each grouped by some feature.

The online resource MENAP [41,42,43] was used to model the ecological network of urban dust bacterial communities in Moscow. The results were visualised using Cytoscape 3.10.3 software [44].

## 3. Results

### 3.1. Biodiversity and Taxonomic Characterization of the Bacterial Community of Dust Particles

A total of 3632 amplicon sequence variants (ASVs) for urban dust bacterial communities (Table S2) were detected after metabarcoding and subsequent bioinformatic analysis.

**Figure 1:**
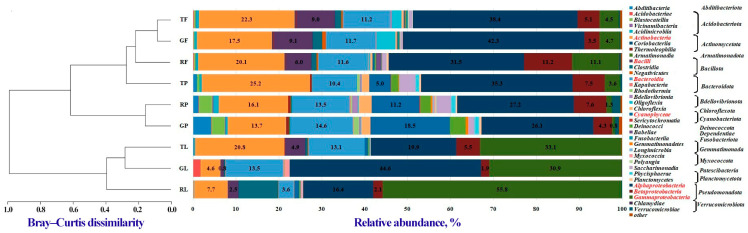
Distribution of the most abundant bacterial classes identified in functional zones and biotopes of Moscow. Classes with relative abundance ≤ 0.1% were considered as “other”. The clustering analysis of the class-level communities based on the calculation of the pairwise Bray–Curtis dissimilarity matrix is also reported. The first letter of the abbreviation refers to the functional zone: T—traffic, R—residential, and G—recreational; the last letter refers to the biotope: L—leaves, P—paved surface, and F—PM10 air.

A total of 29 bacterial phyla were identified in dust samples collected from all functional zones, with the highest relative abundances found for the phyla *Pseudomonadota* (31.2–77.3%), *Bacteroidota* (3.6–16.0%), and *Actinomycetota* (4.6–26.0%) (Table S2). It is noteworthy that the relative abundances of the phyla *Abditibacteriota* (0.9–4.2%), *Acidobacteriota* (0.7–3.6%), *Cyanobacteriota* (5.0–18.5%), *Planctomycetota* (1.4–3.7%), and *Deinococcota* (1.8–3.8%) in the bacterial communities of paved dust from all functional zones were higher than those observed in the phylloplane and air dust particle communities. The relative abundance of the phylum *Bdellovibrionota* (0.5–4.5%) was higher in microbial communities of airborne dust particles compared to bacterial communities of selected phylloplane and paved dust particles. The bacterial communities of paved dust were characterized by the lowest abundance of the phyla *Bacillota* and *Fusobacteriota* (0.1–0.2% and 0%, respectively). For the phylum *Actinomycetota*, the impact of the anthropogenic load was observed (abundance decreased in the direction “traffic zone—residential zone—recreational zone”) and did not depend on the biotope type. It should be noted that paved dust was the most indicative of the influence of different levels of anthropogenic pollution on phyla abundances in bacterial communities. For example, the abundances of the phyla *Pseudomonadota* and *Patescibacteria* in the communities of this biotope decreased with the reduction in the anthropogenic pollution, while the abundance of *Abditibacteriota*, *Bacteroidota*, *Cyanobacteriota*, *Deinococcota*, and *Planctomycetota* increased. It should be noted that the dendrogram constructed on the basis of Bray–Curtis indices to study the differences between the communities did not show any clear clustering at the phylum level, neither by zones nor by biotopes. Nevertheless, clustering by biotope was already evident at the class level (Figure 1). The role of biotopes in the formation of bacterial communities was confirmed by NMDS and PCoA performed at the species level (PERMANOVA; *p*-value = 0.009) (Figure S2).

A total of 69 bacterial classes were identified in the microbial communities of dust particles that were the subject of this investigation (Figure 1). The most prevalent classes were *Actinobacteria* (4.6–25.2%), *Bacteroidia* (3.6–14.6%), *Alphaproteobacteria* (16.4–44.6%), *Betaproteobacteria* (1.9–7.6%), and *Gammaproteobacteria* (0.8–55.8%). The relative abundance of *Gammaproteobacteria* in the microbial communities of leaf dust was found to be more than five times higher than that observed for the bacterial communities of air dust and paved dust. The opposite phenomenon was observed in the class *Gemmatimonadetes*, with a difference exceeding a 10-fold increase. The relative abundances of the *Saccharimonadia*, *Deinococci*, and *Cyanophyceae* classes were found to be higher in the paved dust bacterial communities than in microbial communities of leaf and air dust. In the air dust samples, the highest relative abundance (6.0–9.1%) was found for the class *Bacilli*. 

Figure S3 presents data on the relative abundance of orders in the bacterial communities of the dust particles under study. Significantly, the bacterial communities of paved dust were characterised by the prevalence of the orders *Abditibacteriales*, *Cyanobacteriales*, *Deinococcales*, *Saccharimonadales*, *Rhodobacterales*, and *Sphingomonadales*. The microbial communities of air dust were predominantly represented by members of the orders *Micrococcales*, *Lactobacillales*, *Chitinophagales*, *Rhizobiales*, and *Salinisphaerales*, while the lowest relative representation was observed for *Acetobacterales*. More than 30% of the leaf dust bacterial communities were *Enterobacterales*. No regularities were found in the distribution of taxa according to functional zones of the city.

Figure S4 presents Venn diagrams to show the similarity of bacterial populations identified to the level of genus ASVs. As shown in Figure S4A, the bacterial communities of particulate matter from air samples were markedly different from those of dust from leaves and paved surfaces. This was reflected in the higher number of unique elements observed in the PM10 bacterial communities, which accounted for 25.8% to 41.2% of all identified amplicon sequence variants (ASVs) at the genus level. Furthermore, in each functional zone, the relative abundance of unique elements (ASVs) in air dust was more pronounced than the abundance of common elements between dust from other biotopes. Additionally, the number of shared elements between different biotopes in the residential and traffic zones (areas with a constant anthropogenic chemical pollutant presence) was greater than in the recreational zone (Figure S4B). A comparison of the same biotopes across the three functional zones revealed a prevalence of common elements over unique elements, with the latter being particularly evident in only two zones.

**Figure 2:**
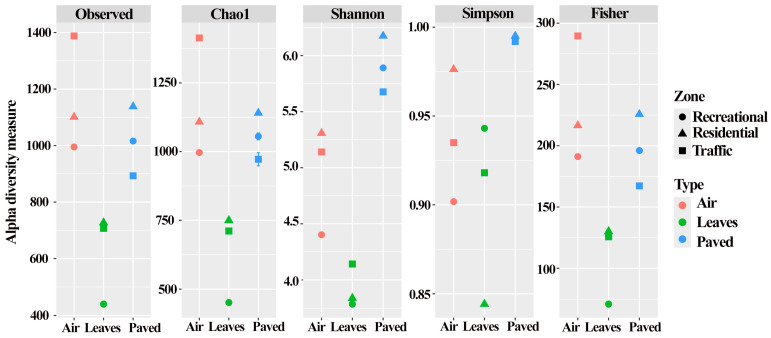
Alpha diversity indices of bacterial communities of dust particles from air, leaf surfaces, and asphalt surfaces collected in three functional zones of the city of Moscow.

In our work, we compared alpha diversity indices to characterise the communities studied. The differences between the alpha diversity indices chosen for comparison (Chao1, Fisher, Shannon, Simpson, and Observed) are that each focuses on different aspects of this concept. For the sake of clarity, the data are presented in two different formats. Figure 2 shows the values individually for each sample.

At the same time, in Figure S5, we present a comparison of alpha diversity indices between bacterial communities of different biotopes with the indication of significance coefficients. In general, the data show that the bacterial communities of leaf dust exhibited low values for the indices mentioned above. Statistically significant differences were found only for the Chao1, Fisher, and Shannon indices (Figure S5). This indicates that the bacterial communities of leaf dust are not highly complex and are characterised by a low species diversity and low species richness. 

Figure 2 shows how the biodiversity indices compare with each other for all the individual communities studied. It was observed that bacterial communities of air PM10 and leaf dust from the recreational zone were characterised by low values of the calculated indices (with the exception of the Simpson index of leaf dust communities). In the case of paved dust, such an effect was observed for the communities of the traffic zone. It should be noted that the bacterial community of paved dust in the residential zone was characterised by high species richness, species diversity, and structural complexity. The high Simpson index in the bacterial communities of air and paved dust in the residential zone indicated the dominance of one or several species.

### 3.2. Functional Characterisation of the Bacterial Community

**Figure 3:**
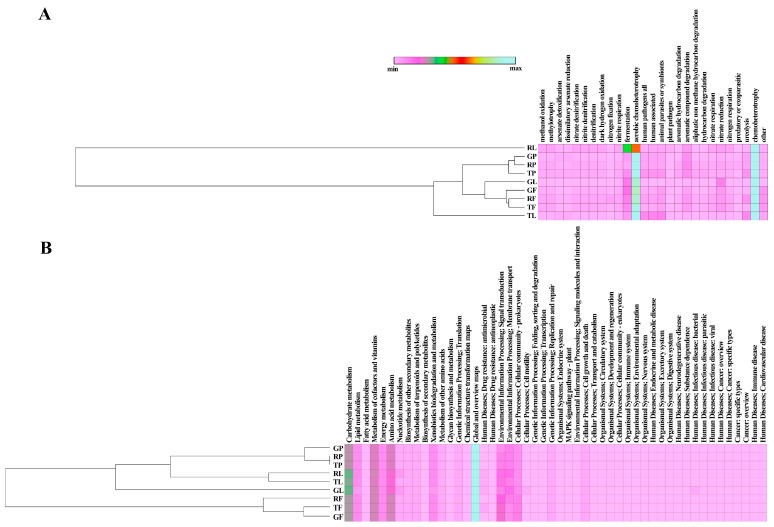
Predicted functional profiles for the bacterial community from FAPROTAX and PICRUSt. (**A**,**B**) Histograms show the predicted functional categories in the bacterial communities from dust.

The FAPROTAX database (Figure 3A) predicted the presence of 53 functions in the studied bacterial communities with at least one record. 

It should be noted that there were only 27 predicted functional traits for which the relative abundances in the communities were higher than 0.01. Among all detected functions, the relative abundances of chemoheterotrophy and aerobic chemoheterotrophy were the highest in all studied samples (33.7–44.1% and 22.1–37.8%, respectively). It was interesting to note that in the predicted functional characteristics of the residential leaf dust bacterial communities, the relative abundances of fermentation and aerobic chemoheterotrophy were also high (13.9% and 22.1%, respectively) compared to these values for the other eight communities. The latter was reflected in the assignment of this bacterial community by function to a separate branch (Figure 3A) when calculating Pearson’s linear correlation coefficients and the subsequent clustering of samples. Also, correlation analysis demonstrated that the bacterial communities of paved dust and air dust particles were differentiated into two distinct functional clades by biotope type, in contrast to the predicted functions of leaf-surface dust communities. The latter indicates that the functional characteristics of these three communities differed significantly from each other. Functions such as human pathogens pneumonia and human pathogens nosocomial were found in bacterial communities of leaf-surface dust and PM 10 air sampled only in the residential and traffic areas. A correlation was found between the functional zoning of the city and the bacterial communities of paved dust. This was reflected in an increase in the relative representation of the functions aromatic hydrocarbon degradation, aromatic compound degradation, aliphatic non-methane hydrocarbon degradation, and hydrocarbon degradation in the direction of increasing anthropogenic impact: from the recreational zone to the residential zone to the traffic zone. This was probably due to an increase in the number of hydrocarbon-oxidising bacteria within the community composition, which occurred in line with the specified direction of the functional zoning of the city.

Figure 3B shows the predicted functions of bacterial dust communities from the PICRUSt database. 50 functional characteristics were detected, with maximum relative abundances of carbohydrate metabolism (9.1–10.4%), lipid metabolism (2.9–3.8%), metabolism of cofactors and vitamins (8.1–8.5%), energy metabolism (3.3–3.4%), amino acid metabolism (7.2–8.6%), nucleotide metabolism (1.8–2.0%), biosynthesis of other secondary metabolites (1.4–1.5%), metabolism of terpenoids and polyketides (1.3–1.6%), xenobiotics biodegradation and metabolism (2.7–3.8%), metabolism of other amino acids (1.4–1.5%), genetic information processing/translation (1.1–1.3%), global and overview maps (31.2–32.5%), environmental Information Processing/membrane transport (3.5–5.9%), environmental information processing/signal transduction cellular processes (4.0–7.9%); cellular community—prokaryotes (3.5–4.9%), genetic information processing/replication and repair (1.1–1.4%), and cellular processes/cell growth and death (1.1–1.3%). Cluster analysis of the results obtained from the PICRUSt database (Figure 3B) also revealed distinct clades of bacterial communities formed by biotopes (namely paved dust and air dust particles), which correlated with the FAPROTAX data (Figure 3A). 

The relative abundance of the module xenobiotics biodegradation and metabolism was found to be lower in leaf dust samples (2.7–3.2%) compared to other types of dust samples. However, there was a positive relationship with functional zones in terms of the increase in the upward direction of anthropogenic pollution in the city. The opposite effect was observed for this type of sample with respect to the indicators of the module human diseases/infectious disease: bacterial. 

It should be noted that the predicted abundance of the module human diseases/ drug resistance: antimicrobial in terms of the functional characterization of the bacterial community of paved dust was lower compared to these values obtained for leaf and air dust communities. We also predicted a statistically significant functional profile of the communities with respect to the presence of antimicrobial-resistance genes using PICRUSt2 2.5.0 software (Table S3). These genes were combined into several modules, and four modules were identified: resistance to beta-lactams (including carbapenems), tetracyclines, cationic antimicrobials, and an efflux module of multidrug resistance systems. Each module consisted of several elements (submodules). When comparing the data obtained as a result of predicting the functional profiles of communities in dust from different types of samples, the highest abundances in gene submodules were in the case of the bacterial community of air dust. However, when we summarised the representation of antibiotic resistance genes in all biotopes, the index decreased significantly in the leaf–air–soil biotopes. Interestingly, the abundances of tetracycline and carbapenem resistance genes was more than 10 times higher in the air bacterial community compared to the paved bacterial community. The abundance of beta-lactam antibiotic resistance module genes decreased significantly in the paved–air–leaf succession, while the opposite effect was found for the abundance of tetracycline resistance module genes. In general, there was a relationship between the presence of predicted functions of antibiotic resistance genes and biotope type. Functional urban zoning did not have a statistically significant effect on the functional profile of bacterial communities of predicted antibiotic-resistant genes.

### 3.3. Characterisation of Antibiotic-Resistant Bacterial Genera of the Cultured Part of the Bacterial Community

The culturable part of the bacterial community was analysed for the presence of antibiotic-resistant members. A range of antibiotics from different classes were used to achieve this, including beta-lactams (ceftazidime, cefepime, and meropenem), tetracyclines (tetracycline), amphenicols (chloramphenicol), and aminoglycosides (streptomycin, kanamycin, gentamicin, and amikacin). The total abundance of heterotrophs in air PM10 was found to be more than twofold lower than in leaf and paved dust (see Figure S6). In this study, 604 isolates were obtained. The studied isolates were characterised by morphological features and their ability to grow on the tested antibiotics as well as by using genomic fingerprinting. As a result of these manipulations, isolates obtained from one dust sample and possessing the same fingerprint, colony morphology, and antibiotic resistance were assigned to one strain or closely related strains of the same species. Seventy antibiotic-resistant strains were selected and identified to genus level. It should be noted that only 27% of the identified strains were capable of growth on only one of the antibiotics tested (Table S1). 

The screening for individual antibiotics demonstrated the presence of bacteria resistant to ceftazidime, cefepime, and tetracycline in the majority of samples (Figure S7). Bacteria resistant to meropenem and amikacin were found only in leaf dust from the residential and traffic zones. Chloramphenicol-resistant strains were identified in leaf and paved dust samples. In general, the proportion of culturable antibiotic-resistant bacteria among heterotrophic organisms ranged from 0.03% to 1.88%, with the highest prevalence observed in leaf dust from the residential zone. It is interesting to note that in the bacterial community of leaf dust, multidrug-resistant strains were found, whose members were resistant to all antibiotics tested (Table S1). 

The identification of bacteria grown on individual antibiotics was achieved by determining the 16S rRNA gene sequence. The identified bacteria were assigned to the genera *Acinetobacter*, *Arthrobacter*, *Bacillus*, *Brachybacterium*, *Brevibacterium*, *Chryseobacterium*, *Chryseomicrobium*, *Methylorubrum*, *Microbacterium*, *Micrococcus*, *Microvirga*, *Pseudarthrobacter*, *Rahnella*, and *Rothia* (Table S4). The antibiotic-resistant strains of *Brevibacterium*, *Rahnella*, and *Chryseomicrobium* were isolated exclusively from PM10 air samples across all functional zones. In contrast, *Pseudarthrobacter* and *Bacillus* were recovered from the majority of leaf and paved dust samples. Additionally, *Acinetobacter* and *Chryseobacterium* were observed in not only PM10 air but also in dust from the surface of leaves in the traffic zone and residential zone, respectively. 

Analyses of the relative representation of the genera of detected culturable antibiotic-resistant bacteria (Table S5) in the dust microbiomes showed that *Brevibacterium* and *Rahnella* were present only in air PM10, which correlates with the data in Table S4. It should be noted that in the bacterial communities of leaf dust from the recreational zone, the relative abundances of detected genera of culturable antibiotic-resistant strains were lower than in zones characterised by anthropogenic pollution.

### 3.4. The Ecological Network of Bacterial Communities in Urban Dust

**Figure 4:**
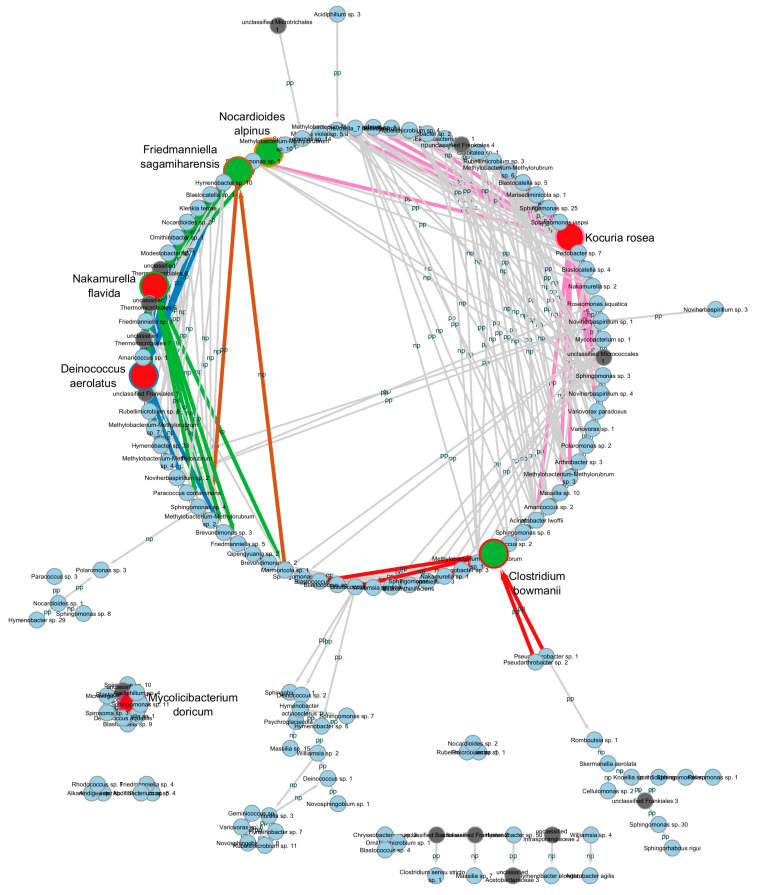
Network visualisation showing the interaction between different bacterial ASVs at the species level (R^2^ = 0.810, *p*-value = 0.039). Large-diameter circles are species with the maximum number of connections.

The Molecular Ecological Network Analyses Pipeline [41] was used to evaluate ecological networks of identified bacteria in microbial communities. The connectivity of each node was determined based on intra-module connectivity (Zi) and inter-module connectivity (Pi) [45]. Potential key elements were determined based on Zi and Pi values with thresholds above 2.2 and 0.56, respectively [46]. Figure 4 shows the ecological network of interaction of bacterial species in all investigated bacterial communities of dust particles found in Moscow in the three functional zones. This includes a total of 148 ASVs (out of 3632 identified in all communities) organised into 16 modules, with 10 modules having no inter-module interactions (Table S6). A total of 352 links were identified in the constructed ecological network, of which almost half (164) were negative interactions. *Deinococcus aerolatus*, *Kocuria rosea*, *Mycolicibacterium doricum*, and *Nakamurella flavida* are intramodule hubs; i.e., they have the maximum number of neighbours within their modules. These intramodule hubs have a mostly positive type of interaction with the bacteria of their module. *Clostridium bowmanii*, *Friedmanniella sagamiharensis*, and *Nocardioides alpinus* showed higher connections outside their modules; i.e., they act as connectors for communication with other modules.

The genera/species of microorganisms found in the ecological network were screened for opportunistic bacteria or those associated with bacteremia and inflammatory processes. Representatives of 26 opportunistic bacterial genera and 30 microorganisms associated with bacteremia or inflammatory processes were found in the ecological network. The first, opportunistic bacteria, had 114 relationships with other microorganisms. The second group had 91 interactions. Thus, more than 200 connections in the constructed ecological network were with bacteria that were dangerous or potentially dangerous to humans. 

*Kocuria rosea* is an opportunistic microorganism [47]. This bacterium was also one of the intramodular centres. Nineteen relationships of *Kocuria rosea* were detected, nine of which were relationships with opportunistic microorganisms, and six of these were expressed as positive effects on these opportunistic microorganisms. This module also includes representatives of the genera of the culturable antibiotic-resistant strains detected: *Acinetobacter*, *Arthrobacter*, and *Methylorubrum*. The representatives of *Pseudarthrobacter* involved in the ecological network were shown in a common module with *Clostridium bowmanii* and had a positive effect on it. As for the genera *Acinetobacter*, *Arthrobacter*, *Pseudarthrobacter*, and *Methylorubrum*, about 25% of all connections of the constructed ecological network were related to interactions with them. Some of the *Pseudarthrobacter* and *Methylorubrum* found in the ecological network were connectors for communication with other modules. A significant number of interactions, both positive and negative, were found between the genera of the culturable antibiotic-resistant strains detected. 

## 4. Discussion

Antimicrobial resistance is a significant global threat to public health on a worldwide scale. The widespread use of antimicrobial drugs serves to accelerate the evolution and selection of naturally occurring antimicrobial-resistance genes in bacteria [48]. There is a considerable amount of research related to the prevalence of antibiotic-resistant strains and genes in natural research subjects [7,8,9,10]. At the same time, the last decade has seen a remarkable increase in research examining air samples in this context [49,50,51,52,53]. This is due to the active development of omics approaches, which allow the assessment of the characteristics of the non-culturable part of the microbial community to be assessed. It is important to study the microbial communities of different environmental objects in the context of antibiotic resistance because soil, water, air, and other media can serve as extensive reservoirs for antibiotic-resistant genes that accumulate over time. Most comprehensive studies of antibiotic resistance within microbial communities in different environmental matrices [49] have not compared this attribute with the microbiomes of dust deposited on the surface of plants (phyllosphere). However, it is known that antibiotics produced by plants and microorganisms in the rhizosphere or endosphere of plants can stimulate the production of antibiotic-resistant genes in the environment [54].

In the framework of the present work, a comprehensive study was conducted to compare bacterial dust communities and their potential antibiotic resistance in different types of biotopes (air, leaves, or paved surfaces), taking into account different levels of anthropogenic load. Our study was carried out on the example of the urban ecosystem of functional zones of Moscow in summer (June). The selection of the season was influenced by the observation that, according to several researchers, bacterial communities in urban biotopes are more heterogeneous in the summer months than in other seasons [55]. In contrast, according to [22], the distribution of antibiotic-resistant genes in the air is not influenced by such a factor as seasonality.

Harnpicharnchai et al. [56] showed remarkable correlations between bacteria containing antibiotic resistance genes and those containing xenobiotic degradation genes. These correlations suggest that the selective pressure exerted by certain xenobiotics and antibiotics may simultaneously affect both antibiotic resistance genes and xenobiotic degradation genes in the environment. Thus, it can be assumed that under conditions of negative impact of xenobiotics, this should lead to the simultaneous development in communities of both microorganisms capable of degrading these toxicants and bacteria resistant to antimicrobial drugs. Such correlations may be due to mechanisms of resistance common to both xenobiotics and antibiotics (reduced rate of entry of compounds into the cell, use of active transport systems for their removal, target modification, etc.). Another common feature of xenobiotic degradation genes and antibiotic resistance genes is that they can be part of mobile genetic elements. They allow the community to adapt quickly to changing environmental conditions. Vetrova et al. [57] showed that the level of PAHs (one of the most toxic types of xenobiotics) varies in different urban functional zones. It was therefore suggested that the quantity of antibiotic-resistant microorganisms is increasing in the zones studied. Pathogenic and opportunistic microflora are of major concern in terms of antibiotic resistance, so we analysed the impact of anthropogenic load on the relative representation of these microorganisms among the identified species and genera in the microbiome. In the present work, with the combination of the approaches used to characterise bacterial communities, no correlation was found with the functional zoning of the city. Minor dependencies on zoning were found only for some taxa and only at a certain level. For example, the relative abundance of the pathogenic bacterial genus *Salmonella* was only detected in microparticles in the traffic area. And the abundance of the genus *Roseomonas*, some species of which can cause bacteremia in humans, in the microbiome was influenced by the level of anthropogenic load (the highest number was observed in the traffic zone).

It should be noted that in the microbiomes of air and leaf-surface dust, a higher percentage of relative abundances of the bacterial genera *Bacillus*, *Enterococcus*, and *Pseudomonas*, among whose representatives are opportunistic microorganisms, was observed compared to their relative abundance in paved dust samples (regardless of the functional zone of the city). The presence of some pathogenic bacterial genera (*Shigella*, *Escherichia*, *Staphylococcus*, *Neisseria*, *Streptococcus*, and *Haemophilus*) was also found only in air and leaf dust microbiomes. The abundance of the abovementioned pathogens in the air dust bacterial community was 2–10 times higher than in the leaf dust microbiome. Despite the fact that the values of the alpha diversity indices of the bacterial communities of air and leaf dust microparticles differed significantly (Figure S4), these communities were the most representative for the detection of opportunistic and pathogenic microflora. It can be suggested that the bacterial community of leaf dust could be used not only to assess air quality but also as an indicator of pathogens in the atmosphere. The above hypothesis was indirectly supported by the results obtained using the FAPROTAX database, which revealed that the microbiomes of air and leaf dust samples collected in residential and traffic areas exhibited a characteristic feature, namely the presence of human pathogens. This phenomenon can be correlated with the fact that these areas have the highest population density. Undoubtedly, the presence of opportunistic microorganisms, including those carrying antibiotic resistance determinants, in the microbiome of environmental objects, especially in the microbiome of airborne dust particles, is a serious public health concern [22,58].

The presence of antibiotic-resistant microorganisms suggests the possibility of transfer of antibiotic resistance determinants between bacteria in the air [59] and can lead to prolonged respiratory illness. In the present study, we performed a statistically significant analysis of the functional profile of bacterial communities to determine the presence of antimicrobial-resistance genes using the PICRUSt database. The most notable finding was the observation that, in the majority of cases, the representation of genes in the bacterial communities of air–leaf–paved biotopes showed a significant decrease compared to the total number of genes included in each individual KEGG module (Table S3). This finding is in accordance with the data presented by Xie J et al. [49], which indicated that the highest diversity of antibiotic resistance genes was observed in air samples from Beijing, in comparison to other samples. It should be noted that when all the antibiotic resistance genes identified by us were summarised using the KEGG module database, a tendency towards a decrease in the representation of bacterial communities in the leaf–air–paved biotopes was observed. It seems plausible to suggest that this may be due to the fact that some microorganisms associated with phylloplane are able to synthesise antibiotics (e.g., streptomycin and penicillin). Furthermore, they can become endophytic bacteria when entering into closer symbiotic relationships with plants [60]. The resulting antibiotics can exert selective pressure on the microbial community, rendering susceptible microorganisms resistant. This phenomenon has been observed in numerous studies [61,62]. Plants are capable of synthesizing a range of antibiotics commonly referred to as phytoalexins, which are employed to inhibit the growth of pathogens [63]. For example, Pedras and Ahiahonu [64] indicated that as a consequence of co-evolution with plants, some pathogens have developed resistance to antibiotics produced by host plants. A third potential mechanism for the emergence of antibiotic-resistant strains within the plant phyllosphere is associated with the filtration of airborne bioaerosols by leaves. This phenomenon is particularly noticeable in summer, when high temperatures cause air currents to flow downwards over green areas. This is due to the fact that the surface of the leaves is much cooler than the paved surfaces of urban roads. Dust carried by the downward air currents settles on the leaves and introduces microorganisms from the air aerosol into the phylloplane surface community. In addition to the biogenic fraction, toxic chemical compounds (e.g., PAHs) affect the microflora of the phylloplane surface together with the dust. However, we suggest that in urban ecosystems, there are many factors (in addition to the presence of certain levels of PAHs and other hydrocarbons) that can influence bacterial communities. Our assumption is based on the absence of a positive correlation in leaf dust communities between the relative abundances of the xenobiotics biodegradation and metabolism and human diseases; drug resistance: antimicrobial modules, according to PICRUSt data. At the same time, the PICRUSt data indicate that the main potential reservoirs of antimicrobial-resistance genes are microorganisms present in the air aerosol and on the leaf surface.

In the present study, an attempt was also made to assess the resistome of the culturable bacterial communities present in the dust samples with respect to resistance to different groups of antibiotics, namely tetracyclines, aminoglycosides, β-lactams, and amphenicols. The majority of the isolates were found to be resistant to tetracycline and β-lactams, which correlated with the results reported by several researchers [65]. Furthermore, Wu et al. [18] concluded that genes encoding resistance to aminoglycosides, macrolides, lincosamides, streptogramines, tetracyclines, and β-lactams represent the primary components of the resistome of urban dust samples. Rosas et al. [14] found multidrug-resistant isolates in the environment. This also correlated with our data since more than 70% of the isolates obtained in this study were resistant to several antibiotics, and almost 43% were resistant to antibiotics belonging to different chemical groups. The latter suggests that the presence of antibiotic resistance determinants in the microbiomes of urban ecosystems and their potential distribution need to be studied in detail. In this context, the analysis of microbial communities should include not only the search for opportunistic microorganisms but also the assessment of interactions between members of the microbial community.

As a result of constructing an ecological network of microorganism interactions between all the biotopes studied, it was found that more than one-third of the microorganisms that formed its basis were opportunistic bacteria and inflammatory microorganisms. Importantly, their branching relationships accounted for almost two-thirds of all interactions. This again suggests that the urban ecosystem is one of the potential sources for the development and spread of human diseases. Interestingly, abundances of the genera *Arthrobacter*, *Acinetobacter*, *Methylorubrum*, and *Pseudarthrobacter*, to which some isolated antibiotic-resistant strains belonged, were also included in the predicted ecological network of the bacterial community, acting both as intramodular hubs and as connectors for communication with other modules. In other words, the predicted network included not only potential sources of disease but also likely ‘donors’ and ‘recipients’ of antimicrobial-resistance determinants. For example, according to our data, among the identified culturable antibiotic-resistant bacteria, the largest number of isolated strains belonged to the genus *Pseudarthrobacter*. It is noteworthy that the majority of isolated *Pseudarthrobacter* strains had multidrug-resistant properties. These properties can be transferred to closely related microorganisms, such as bacteria of the genus *Arthrobacter* [66]. This will lead to the spread of resistance genes in the community.

The results obtained indicate that it is important to study the microbiome of urban dust, especially air, not only from the point of view of ecological–geological assessments of the urban ecosystem but also from the point of view of assessing the threat of the mutual interaction of antibiotic-resistant representatives of these communities with clinically important bacterial strains.

## 5. Conclusions

Air pollution plays a role in the proliferation of antibiotic-resistant bacteria in the microbiome of dust particles, which is a major public health concern. A review of the scientific literature on metagenomic and functional studies indicates that antibiotic resistance genes are present in all microbial communities. It is of particular importance to analyse the genetic determinants of bacterial communities associated with antibiotic resistance among airborne microorganisms associated with PM particles due to their ability to disperse over long distances and circulate in both outdoor and indoor environments. The present study characterized the structure and functionality of bacterial communities of dust particle samples collected in three biotopes of urban functional zones of Moscow and compared the prevalence of the predicted resistome modules and culturable antibiotic-resistant bacteria in microbiomes. A key finding was that the ecological network of the microbial community of an urban ecosystem as a whole contained and interacted with representatives of both opportunistic bacteria and bacteria associated with bacteremia and inflammation as well as potential ‘donors’ and ‘recipients’ of antibiotic resistance determinants. The study of the microbiome and resistome as a whole has the potential to expand our understanding of the distribution of antibiotic resistance genes and the mutual exchange of these genes within different microbial communities in natural and urban ecosystems.

## Data Availability

The dataset is available on the NCBI SRA Portal; the accession numbers are PRJNA1142405 and PRJNA928599 (bacterial communities).

## Supplementary Materials

The following supporting information can be downloaded at https://www.mdpi.com/article/10.3390/biology13121022/s1: Figure S1. Overview of the three urban sites (Traffic, Residential, and Recreational zones) of Moscow; Figure S2. Non-metric multidimensional scaling (NMDS) (A) and principal coordinate analysis (PCoA) (B) of bacterial communities; Figure S3. Distribution of the most abundant bacterial classes (relative abundance > 0.1%) identified in functional zones and biotopes of Moscow. Classes with relative abundance ≤ 0.1% were considered as “other”. The clustering analysis of the class-level communities based on the calculation of the pairwise Bray–Curtis dissimilarity matrix is also reported. The first letter of the abbreviation refers to the functional zone: T—traffic, R—residential, and G—recreational; the last letter refers to the biotope: L—leaves, P—paved surface, and F—filter/PM10 air; Figure S4. Venn diagrams illustrating the number of unique and shared bacterial ASVs at the level of genus among dust particles from Moscow that were detected in three urban zones; Figure S5. Comparisons of Chao1 richness, Fisher, and Shannon diversity of bacterial communities between Moscow biotopes. Horizontal lines indicate significance levels between groups (* *p* < 0.05, *** *p* < 0.001); Figure S6. Numbers of heterotrophs in the bacterial communities of air microparticles, leaf, and paved dust; Figure S7. Total number of antibiotic-resistant bacteria grown on individual antibiotics from total heterotrophic bacteria. Data are shown as percentages; Table S1. Antibiotic resistance and identification of typical isolated colonies of cultured strains; Table S2. Relative abundance of bacterial ASVs by level phylum, class and order; Table S3. Total abundances of genes included in the respective KEGG modules (*p* < 0.05); Table S4. Representation of genera of culturable antibiotic-resistant strains in the bacterial communities of selected dust particles; Table S5. Relative abundance of genera of culturable antibiotic-resistant strains in the bacterial communities of selected dust particles; Table S6. Cytoscape data for network visualisation showing the interaction between different bacterial ASVs at the species level in dust samples.
