# Supplementary material for: Antibiotic Resistance as a Functional Characteristic of Urban Dust Particles’ Microbial Communities
Source: Biology (Basel). 2024 Dec 6;13(12):1022. doi: 10.3390/biology13121022 (PMC11672966; doi:10.3390/biology13121022)
Supplement: Supplementary file 1 [file biology-13-01022-s001.zip › Supplementary materials_24.11.24.pdf]

Supplementary materials.

Antibiotic resistance as functional characteristic of urban dust particles

Anna A. Vetrova[1,*], Anastasia A. Ivanova[1], Kirill V. Petrikov[1], Olga Gavrichkova[2], Maria V. Korneykova[3,4] and Olesya I. Sazonova[1]

[1]Federal Research Center "Pushchino Scientific Center for Biological Research of the Russian Academy of Sciences", 142290 Pushchino, Russia; sazonova_oi@rambler.ru (O.I.S.), mrs.ivanova.a.a@gmail.com (A.A.I.), bioscience.kp@gmail.com (K.V.P)
[2] Research Institute on Terrestrial Ecosystems, National Research Council, 05010 Porano, Italy, olga.gavrichkova@cnr.it (O.G.)
[3] Agrarian and Technological Institute, Peoples' Friendship University of Russia (RUDN University), 117198 Moscow, Russia; korneykova.maria@mail.ru (M.V.K)
[4] Institute of North Industrial Ecology Problems Subdivision of the Federal Research Center "Kola Science Centre of Russian Academy of Science", 184209 Apatity, Russia
*Correspondence: phdvetrova@gmail.com (A.A.V.)

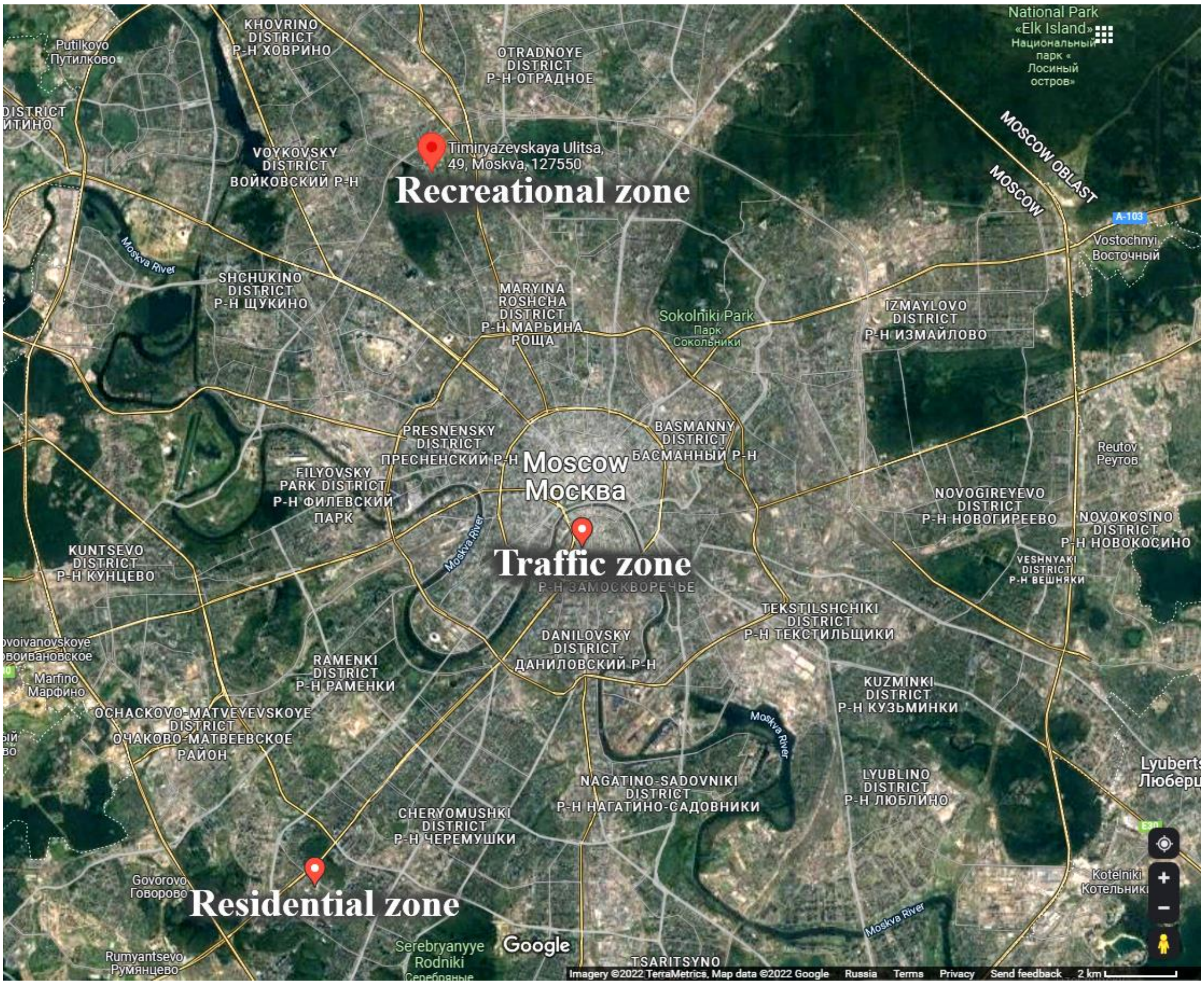

Figure S1: Overview of the three urban sites (Traffic, Residential and Recreational zones) of Moscow

Table S1. Antibiotic resistance and identification of typical isolated colonies of cultured strains

Strains	Antibiotic resistance	Genus identification
TP 16	Cfz, Gm 40, Gm 10, Tc	*Microbacterium*
TP 2, TP 20, TP 17	Cfz, Cf, Gm 10	*Pseudarthrobacter*
TP 25	Cfz, Cf, Gm 10	*Bacillus*
TP 23, TP 27, TP 29	Cfz, Gm 10, Gm 40	*Microbacterium*
TP 18	Cfz	*Pseudarthrobacter*
TP 37	Tc	*Brachybacterium*
RP 17	Cfz, Cf, Tc	*Bacillus*
RP 2, RP6	Cfz	*Pseudarthrobacter*

RP 1, RP 8, RP 21	Cfz, Cf	*Pseudarthrobacter*
RP 7	Cfz	*Arthrobacter*
RP 4	Cfz, Ch, Km	*Pseudarthrobacter*
RP 1	Cfz, Cf	*Arthrobacter*
GP 46, GP 47	Cfz, Gm 10	*Pseudarthrobacter*
GP 36, GP 37, GP 34, GP 35	Cfz	*Pseudarthrobacter*
GP 39, GP 43, GP 41	Cfz, Cf, Gm 10	*Pseudarthrobacter*
GP 45, GP 48	Cfz, Cf	*Pseudarthrobacter*
TL 25	Cfz, Cf	*Pseudarthrobacter*
TL 26	Cfz, Gm10, Tc, Km, Sm	*Acinetobacter*
TL 29	Tc, Gm 10	*Rothia*
TL 30	Tc, Sm, Mr	*Bacillus*
TL 24	Gm 10, Gm 40, Km	*Microvirga*
TL 14	Tc	*Brachybacterium*
RL 1	Cfz, Cf	*Bacillus*
RL 3	Cfz, Cf, Tc, Sm, Km, Mr, Ak, Ch, Gm 40, Gm10	*Pseudarthrobacter*
RL 4	Tc, Mer, Cfz	*Methylorubrum*
RL 5	Tc, Cf	*Bacillus*
RL 6, RL 8	Cf	*Pseudarthrobacter*
RL 7	Cfz	*Pseudarthrobacter*
RL 15, RL 16	Tc	*Chryseobacterium*
RL 12	Gm 10, Gm 40, Km, Sm	*Microvirga*
GL 27	Cfz	*Rothia*
GL 28, GL 14	Cfz, Cf, Km	*Bacillus*
GL 15, GL 17, GL 19	Cfz, Cf	*Pseudarthrobacter*
TF 19, TF 20, TF 21	Sm, Tc, Cfz, Km	*Acinetobacter*
TF 9	Cf	*Chryseomicrobium*
TF 23	Sm	*Brevibacterium*
TF 14	Tc, Cfz	*Brachybacterium*
TF 13	Tc	*Rahnella*
RF 10	Cf	*Chryseomicrobium*
RF 6	Tc	*Rahnella*
RF 3	Tc	*Chryseobacterium*
RF 4	Sm	*Brevibacterium*
GF 11	Cf	*Chryseomicrobium*
GF 12	Tc, Km, Sm	*Acinetobacter*
GF 14	Cfz, Tc	*Chryseobacterium*
GF 17	Cfz, Sm	*Micrococcus*
GF 18	Sm	*Micrococcus*
GF 7	Sm, Km	*Brevibacterium*
GF 5	Tc	*Rahnella*

Table S2. Relative abundance of bacterial ASVs by level Phylum, Class and Order.

Phylum	Class	Order	GP	RP	TP	GL	RL	TL	GF	RF	TF
Abditibacteriota	*Abditibacteria*	*Abditibacteriales*	4,23	1,26	0,91	0,05	0,06	0,36	0,05	0,06	0,09
Acidobacteriota	*Acidobacteriae*	*Acidobacteriales*	0,00	0,00	0,00	1,75	0,10	0,00	0,04	0,02	0,12
		Bryobacterales	0,06	0,10	0,00	0,00	0,00	0,01	0,00	0,14	0,04
		Paludibaculum	0,00	0,01	0,00	0,00	0,00	0,00	0,00	0,00	0,00
		unclassified Acidobacteriae	0,00	0,00	0,00	0,00	0,00	0,00	0,00	0,00	0,01
		unclassified Subgroup 2	0,00	0,00	0,00	0,00	0,00	0,00	0,02	0,00	0,00
	Blastocatellia	*Blastocatellales*	2,93	3,11	0,64	0,04	0,11	0,11	0,19	0,43	0,34
		Pyrinomonadales	0,22	0,03	0,00	0,00	0,00	0,00	0,00	0,00	0,00
		unclassified Blastocatellia	0,00	0,00	0,00	0,00	0,00	0,00	0,00	0,00	0,01
	Holophagae	*Subgroup 7*	0,06	0,07	0,00	0,00	0,00	0,00	0,01	0,02	0,01
	Thermoanaerobaculia	*Thermoanaerobaculales*	0,00	0,00	0,00	0,00	0,00	0,00	0,00	0,00	0,01
	Vicinamibacteria	*Vicinamibacterales*	0,14	0,23	0,03	0,00	0,00	0,00	0,06	0,08	0,10
	Acidimicrobiia	*Microtrichales*	0,43	1,23	0,40	0,02	0,07	0,06	0,56	0,46	0,65
		unclassified Acidimicrobiia	0,05	0,06	0,07	0,01	0,02	0,00	0,01	0,04	0,04
Actinomycetota	*Actinobacteria*	*Actinomycetales*	0,00	0,00	0,00	0,05	0,12	0,12	0,47	0,24	0,32
		Bifidobacteriales	0,00	0,00	0,00	0,02	0,01	0,16	0,04	0,03	0,06
		Corynebacteriales	1,29	2,82	4,20	0,23	0,50	0,55	1,78	3,99	2,91
		Frankiales	4,45	4,56	9,26	0,66	1,71	8,97	1,20	2,52	2,29
		Glycomycetales	0,00	0,00	0,00	0,00	0,00	0,00	0,00	0,00	0,05
		Kineosporiales	0,25	0,18	0,10	0,28	0,16	0,36	0,11	0,13	0,15
		Micrococcales	3,31	3,60	7,23	1,87	2,73	3,86	9,73	9,36	10,60
		Micromonosporales	0,00	0,00	0,00	0,00	0,00	0,00	0,00	0,00	0,02
		PeM15	0,00	0,00	0,00	0,00	0,00	0,00	0,00	0,00	0,01
		Propionibacteriales	4,23	4,89	4,37	1,46	2,48	6,75	3,85	3,49	3,35
		Pseudonocardiales	0,14	0,08	0,02	0,00	0,01	0,01	0,11	0,13	0,90
		Streptomycetales	0,00	0,00	0,00	0,00	0,00	0,00	0,17	0,21	1,20
		Streptosporangiales	0,00	0,00	0,00	0,00	0,00	0,00	0,00	0,02	0,45
		unclassified Actinobacteria	0,00	0,00	0,00	0,00	0,00	0,00	0,01	0,00	0,01
	Coriobacteriia	*Coriobacteriales*	0,01	0,00	0,00	0,02	0,08	0,02	0,21	0,14	0,22
	Rubrobacteria	*Rubrobacterales*	0,00	0,00	0,00	0,00	0,00	0,00	0,00	0,00	0,02
	Thermoleophilia	*Gaiellales*	0,07	0,12	0,04	0,00	0,01	0,02	0,10	0,10	0,04
		Solirubrobacterales	0,56	0,44	0,33	0,00	0,02	0,01	0,11	0,08	0,17
	unclassified Actinomycetota	*unclassified Actinomycetota*	0,00	0,00	0,00	0,00	0,00	0,00	0,00	0,01	0,00
Armatimonadota	*Armatimonadia*	*Armatimonadales*	0,17	0,18	0,10	0,00	0,00	0,00	0,00	0,03	0,00
	Fimbriimonadia	*Fimbriimonadales*	0,00	0,00	0,00	0,02	0,00	0,04	0,00	0,00	0,01
	unclassified Armatimonadota	*unclassified Armatimonadota*	0,00	0,00	0,00	0,00	0,00	0,00	0,00	0,00	0,00
Bacillota	*Bacilli*	*Acholeplasmatales*	0,00	0,00	0,00	0,00	0,09	0,00	0,00	0,00	0,00
		Alicyclobacillales	0,00	0,00	0,00	0,00	0,05	0,01	0,04	0,02	0,03
		Aneurinibacillales	0,00	0,00	0,00	0,00	0,00	0,00	0,00	0,00	0,04
		Bacillales	0,02	0,03	0,03	0,20	0,47	3,53	1,62	1,61	3,78
		Brevibacillales	0,00	0,00	0,00	0,00	0,00	0,00	0,01	0,01	0,04
		Caldalkalibacillales	0,00	0,00	0,00	0,00	0,00	0,00	0,00	0,00	0,01
		Entomoplasmatales	0,00	0,00	0,00	0,00	0,00	0,00	0,05	0,09	0,07
		Erysipelotrichales	0,01	0,00	0,00	0,03	0,25	0,01	0,13	0,05	0,04
		Exiguobacterales	0,00	0,04	0,01	0,03	0,04	0,03	0,08	0,14	0,19
		Haloplasmatales	0,00	0,00	0,00	0,00	0,00	0,00	0,00	0,00	0,01
		Lactobacillales	0,00	0,00	0,00	0,44	1,30	1,12	5,49	3,42	3,38

		Mycoplasmatales	0,00	0,00	0,00	0,00	0,00	0,00	0,00	0,01	0,01
		Paenibacillales	0,00	0,00	0,00	0,02	0,03	0,02	0,10	0,05	0,14
		Staphylococcales	0,00	0,00	0,01	0,07	0,23	0,16	1,52	0,54	1,12
		Thermoactinomycetales	0,00	0,00	0,00	0,00	0,02	0,00	0,04	0,06	0,11
		unclassified Bacilli	0,00	0,00	0,00	0,00	0,00	0,00	0,01	0,00	0,01
	Clostridia	*Caldicoprobacterales*	0,00	0,00	0,00	0,01	0,00	0,00	0,00	0,00	0,00
		Christensenellales	0,00	0,00	0,00	0,00	0,02	0,00	0,00	0,01	0,00
		Clostridia UCG-014	0,00	0,00	0,00	0,00	0,08	0,00	0,06	0,01	0,03
		Clostridia vadinBB60 group	0,00	0,00	0,00	0,00	0,02	0,00	0,00	0,00	0,00
		Clostridiales	0,03	0,08	0,01	0,10	0,26	0,11	0,52	0,40	0,37
		Lachnospirales	0,00	0,00	0,00	0,02	7,91	0,13	0,52	0,25	0,35
		MAT-CR-H4-C10	0,00	0,00	0,00	0,00	0,00	0,00	0,01	0,00	0,00
		Monoglobales	0,00	0,00	0,00	0,00	0,13	0,00	0,00	0,00	0,00
		Oscillospirales	0,00	0,00	0,00	0,04	0,59	0,14	0,48	0,21	0,37
		Peptostreptococcales-Tissierellales	0,03	0,01	0,02	0,04	0,10	0,05	0,58	0,20	0,29
	Desulfitobacteriia	*Desulfitobacteriales*	0,00	0,00	0,00	0,00	0,00	0,00	0,01	0,02	0,01
	Limnochordia	*Hydrogenispora*	0,00	0,00	0,00	0,00	0,00	0,00	0,00	0,00	0,01
	Negativicutes	*Acidaminococcales*	0,00	0,00	0,00	0,00	0,00	0,00	0,01	0,00	0,01
	Negativicutes	*Veillonellales-Selenomonadales*	0,00	0,00	0,00	0,05	0,15	0,14	0,77	0,42	0,39
	Symbiobacteriia	*Symbiobacteriales*	0,00	0,00	0,00	0,00	0,00	0,00	0,00	0,00	0,01
	unclassified Bacillota	*unclassified Bacillota*	0,00	0,00	0,00	0,00	0,00	0,00	0,01	0,00	0,00
Bacteroidota	*Bacteroidia*	*Bacteroidales*	0,02	0,01	0,00	0,11	0,58	0,38	1,41	0,87	1,13
		Chitinophagales	1,51	3,29	0,76	0,07	0,18	0,56	8,09	6,04	6,17
		Cytophagales	9,72	7,59	8,67	4,05	1,92	11,33	0,85	2,60	1,79
		Flavobacteriales	1,79	1,66	0,52	0,05	0,30	0,11	1,07	1,54	1,46
		Sphingobacteriales	1,57	0,91	0,46	9,26	0,58	0,76	0,27	0,52	0,64
		unclassified Bacteroidia	0,00	0,00	0,00	0,00	0,00	0,00	0,01	0,00	0,00
	Kapabacteria	*Kapabacteriales*	0,09	0,15	0,00	0,00	0,00	0,00	0,01	0,00	0,03
	Kryptonia	*Kryptoniales*	0,00	0,00	0,00	0,00	0,00	0,00	0,00	0,00	0,00
	Rhodothermia	*Balneolales*	0,00	0,00	0,00	0,00	0,00	0,00	0,00	0,00	0,01
		Rhodothermales	1,34	0,37	0,68	0,00	0,01	0,02	0,07	0,07	0,05
Bdellovibrionota	*Bdellovibrionia*	*Bacteriovoracales*	0,11	0,31	0,16	0,00	0,02	0,01	0,06	0,13	0,06
		Bdellovibrionales	0,29	1,01	0,19	0,07	0,00	0,02	0,03	0,08	0,08
	Oligoflexia	*Oligoflexales*	0,06	0,16	0,09	0,01	0,00	0,00	0,06	0,09	0,06
		Silvanigrellales	0,09	0,11	0,00	0,00	0,00	0,00	0,00	0,00	0,00
		unclassified Oligoflexia	0,03	0,03	0,00	0,01	0,00	0,00	4,35	0,17	2,27
Campylobacterota	*Campylobacteria*	*Campylobacterales*	0,00	0,00	0,00	0,00	0,00	0,01	0,01	0,00	0,00
Chloroflexota	*Anaerolineae*	*Anaerolineales*	0,00	0,00	0,00	0,00	0,00	0,00	0,00	0,01	0,00
		Ardenticatenales	0,00	0,02	0,00	0,00	0,00	0,00	0,00	0,00	0,00
		Caldilineales	0,01	0,05	0,00	0,00	0,00	0,00	0,02	0,01	0,03
		RBG-13-54-9	0,00	0,00	0,00	0,00	0,00	0,00	0,00	0,00	0,01
		SBR1031	0,01	0,00	0,00	0,00	0,00	0,00	0,00	0,00	0,00
		unclassified Anaerolineae	0,00	0,00	0,00	0,01	0,00	0,00	0,00	0,00	0,00
	Chloroflexia	*Chloroflexales*	0,02	0,04	0,00	0,00	0,00	0,00	0,00	0,01	0,00
		Kallotenuales	0,41	0,47	0,08	0,00	0,00	0,00	0,00	0,00	0,01
		Thermomicrobiales	1,66	2,47	1,67	0,05	0,09	0,04	0,27	0,48	0,46
	Dehalococcoidia	*SAR202 clade*	0,00	0,00	0,00	0,00	0,00	0,00	0,00	0,00	0,01
	Ktedonobacteria	*Ktedonobacterales*	0,00	0,00	0,00	0,00	0,00	0,00	0,02	0,00	0,00
	unclassified Chloroflexota	*unclassified Chloroflexota*	0,01	0,05	0,01	0,02	0,04	0,00	0,13	0,15	0,21

Cyanobacteriota	*Cyanophyceae*	*Cyanobacteriales*	18,48	11,16	4,87	0,07	1,10	0,69	0,09	0,46	0,21
		Leptolyngbyales	0,00	0,00	0,11	0,00	0,00	0,00	0,00	0,00	0,00
		Phormidesmiales	0,00	0,00	0,00	0,00	0,00	0,00	0,00	0,00	0,01
		Synechococcales	0,00	0,00	0,00	0,00	0,01	0,02	0,04	0,02	0,03
		unclassified Cyanobacteriia	0,00	0,00	0,00	0,00	0,00	0,00	0,00	0,00	0,00
	Sericytochromatia	*unclassified Sericytochromatia*	0,03	0,17	0,05	0,00	0,00	0,00	0,03	0,02	0,04
	Vampirivibrionia	*Gastranaerophilales*	0,00	0,00	0,00	0,00	0,00	0,00	0,02	0,00	0,00
		Obscuribacterales	0,00	0,00	0,00	0,00	0,00	0,00	0,00	0,01	0,00
Deinococcota	*Deinococci*	*Deinococcales*	3,83	2,52	1,79	0,04	0,25	0,32	0,14	0,39	0,35
Dependentiae	*Babeliae*	*Babeliales*	0,00	0,00	0,00	0,00	0,00	0,00	0,00	1,13	0,01
Desulfobacterota	*Desulfuromonadia*	*Geobacterales*	0,00	0,00	0,00	0,00	0,00	0,00	0,01	0,01	0,00
Elusimicrobiota	*Elusimicrobia*	*Lineage IV*	0,00	0,00	0,00	0,00	0,00	0,00	0,04	0,00	0,03
Fusobacteriota	*Fusobacteriia*	*Fusobacteriales*	0,00	0,00	0,00	0,01	0,10	0,07	0,37	0,16	0,20
Gemmatimonadota	*Gemmatimonadetes*	*Gemmatimonadales*	0,39	0,26	0,18	0,00	0,00	0,01	0,09	0,07	0,09
	Longimicrobia	*Longimicrobiales*	0,05	0,43	0,07	0,00	0,00	0,00	0,00	0,00	0,00
	S0134 terrestrial group	*unclassified S0134 terrestrial group*	0,00	0,05	0,00	0,00	0,00	0,00	0,00	0,00	0,01
Methylomirabilota	*Methylomirabilia*	*Rokubacteriales*	0,00	0,00	0,00	0,00	0,00	0,00	0,00	0,00	0,01
Myxococcota	*Myxococcia*	*Myxococcales*	0,06	0,29	0,02	1,15	0,37	0,11	0,03	0,04	0,09
	Polyangia	*Haliangiales*	0,02	0,08	0,00	0,00	0,00	0,00	0,01	0,00	0,00
		mle1-27	0,01	0,01	0,00	0,00	0,00	0,00	0,00	0,01	0,01
		Nannocystales	0,02	0,01	0,00	0,00	0,00	0,00	0,00	0,00	0,01
		Polyangiales	0,00	0,16	0,00	0,00	0,00	0,03	0,09	0,04	0,10
Nitrospirota	*Nitrospiria*	*Nitrospirales*	0,03	0,00	0,00	0,00	0,00	0,00	0,00	0,00	0,00
Patescibacteria	*Gracilibacteria*	*Absconditabacteriales (SR1)*	0,00	0,00	0,00	0,00	0,00	0,00	0,01	0,00	0,00
	Parcubacteria	*Candidatus Moranbacteria*	0,00	0,01	0,00	0,00	0,00	0,00	0,01	0,00	0,00
		Candidatus Nomurabacteria	0,01	0,00	0,00	0,00	0,00	0,00	0,01	0,00	0,00
		unclassified Parcubacteria	0,01	0,00	0,00	0,00	0,00	0,00	0,01	0,00	0,00
	Saccharimonadia	*Saccharimonadales*	1,31	3,35	3,66	0,05	0,14	0,08	0,45	0,89	0,82
	unclassified Patescibacteria	*unclassified Patescibacteria*	0,59	0,00	0,00	0,00	0,00	0,00	0,00	0,00	0,00
Planctomycetota	*Phycisphaerae*	*Tepidisphaerales*	1,26	1,15	0,94	0,01	0,04	0,01	0,00	0,09	0,05
	Planctomycetes	*Gemmatales*	0,03	0,09	0,00	0,00	0,00	0,00	0,01	0,00	0,01
		Isosphaerales	0,28	0,15	0,40	0,00	0,03	0,01	0,01	0,46	0,04
		Pirellulales	0,06	0,05	0,00	0,00	0,00	0,00	0,01	0,00	0,04
		Planctomycetales	0,02	0,01	0,01	0,00	0,00	0,00	0,03	0,01	0,01
	vadinHA49	*unclassified vadinHA50*	0,00	0,00	0,00	0,00	0,00	0,00	0,01	0,00	0,00
Pseudomonadota	*Acidithiobacillia*	*Acidithiobacillales*	0,00	0,06	0,02	0,00	0,00	0,00	0,00	0,01	0,03
	Alphaproteobacteria	*Acetobacterales*	5,36	3,81	6,16	16,86	5,06	6,20	0,83	1,35	1,49
		Azospirillales	0,22	0,62	4,28	0,08	0,36	0,19	0,17	0,24	0,19
		Caedibacterales	0,00	0,00	0,00	0,00	0,00	0,00	0,07	0,00	0,00
		Caulobacterales	1,05	0,70	1,53	0,01	0,14	0,13	0,67	3,06	0,61
		Defluviicoccales	0,02	0,14	0,02	0,00	0,04	0,00	0,06	0,06	0,08
		Elsterales	0,00	0,00	0,00	0,00	0,00	0,00	0,00	0,00	0,01
		Ferrovibrionales	0,00	0,00	0,00	0,00	0,00	0,00	0,00	0,00	0,00
		Hyphomicrobiales	0,00	0,00	0,01	19,77	3,65	0,28	0,31	0,32	0,38
		Micavibrionales	0,00	0,00	0,00	0,00	0,00	0,00	0,00	0,00	0,00
		Paracaedibacterales	0,00	0,00	0,00	0,00	0,00	0,00	0,02	0,00	0,01
		Reyranellales	0,01	0,01	0,01	0,00	0,00	0,00	0,00	0,47	0,01
		Rhizobiales	2,66	1,72	3,49	0,66	1,09	2,18	31,30	16,63	26,35
		Rhodobacterales	6,37	8,13	5,03	0,40	2,27	1,24	2,13	3,45	2,98

		Rhodospirillales	0,00	0,00	0,00	0,00	0,00	0,00	0,00	0,01	0,00
		Rickettsiales	0,00	0,00	0,01	0,02	0,03	0,05	0,16	0,12	0,13
		Sphingomonadales	10,25	11,86	14,70	6,76	3,71	9,64	6,58	5,78	6,11
		Tistrellales	0,15	0,22	0,06	0,00	0,03	0,00	0,02	0,03	0,02
	Betaproteobacteria	*Burkholderiales*	4,30	7,57	7,48	1,87	1,98	5,34	2,88	10,81	4,64
		Neisseriales	0,00	0,00	0,00	0,02	0,16	0,10	0,60	0,30	0,39
		Nitrosomonadales	0,01	0,01	0,00	0,00	0,00	0,01	0,04	0,04	0,02
		Rhodocyclales	0,00	0,00	0,00	0,00	0,00	0,00	0,01	0,00	0,01
	Gammaproteobacteria	*Coxiellales*	0,00	0,00	0,00	0,00	0,00	0,00	0,00	0,00	0,00
		Diplorickettsiales	0,00	0,00	0,00	0,00	0,01	0,00	0,01	0,00	0,02
		Enterobacterales	0,00	0,01	0,01	30,18	52,71	30,61	1,09	0,58	0,92
		Gammaproteobacteria	0,00	0,00	0,00	0,00	0,00	0,00	0,01	0,00	0,00
		Gammaproteobacteria Incertae Sedis	0,00	0,00	0,00	0,00	0,00	0,00	0,00	1,98	0,02
		Immundisolibacterales	0,00	0,02	0,00	0,00	0,00	0,00	0,00	0,00	0,00
		Legionellales	0,00	0,02	0,00	0,00	0,00	0,00	0,00	0,01	0,00
		Methylococcales	0,00	0,00	0,00	0,00	0,00	0,00	0,00	0,00	0,01
		Nitrosococcales	0,00	0,00	0,00	0,00	0,00	0,00	0,00	0,01	0,00
		Piscirickettsiales	0,00	0,00	0,00	0,00	0,00	0,00	0,01	0,01	0,00
		Pseudomonadales	0,11	0,77	3,44	0,68	3,01	2,45	2,54	2,24	2,75
		Salinisphaerales	0,00	0,00	0,00	0,00	0,00	0,00	0,46	5,93	0,18
		Steroidobacterales	0,00	0,00	0,00	0,00	0,00	0,00	0,01	0,00	0,00
		unclassified Gammaproteobacteria	0,00	0,00	0,01	0,01	0,00	0,00	0,02	0,00	0,00
		Xanthomonadales	0,66	0,46	0,18	0,01	0,05	0,02	0,51	0,30	0,62
	unclassified Pseudomonadota	*unclassified Pseudomonadota*	0,00	0,01	0,00	0,00	0,00	0,00	0,01	0,00	0,04
RCP2-54	*unclassified RCP2-55*	*unclassified RCP2-56*	0,00	0,00	0,00	0,00	0,00	0,00	0,00	0,00	0,02
SAR324 clade (Marine group B)	*unclassified SAR324 clade (Marine group B)*	*unclassified SAR324 clade (Marine group B)*	0,04	0,00	0,00	0,00	0,00	0,00	0,00	0,00	0,00
Spirochaetota	*Spirochaetia*	*Spirochaetales*	0,00	0,00	0,00	0,00	0,00	0,00	0,01	0,00	0,01
Sumerlaeota	*Sumerlaeia*	*Sumerlaeales*	0,00	0,00	0,00	0,00	0,00	0,00	0,00	0,01	0,00
Synergistota	*Synergistia*	*Synergistales*	0,00	0,00	0,00	0,00	0,00	0,00	0,00	0,00	0,00
Verrucomicrobiota	*Chlamydiae*	*Chlamydiales*	0,02	0,00	0,00	0,11	0,02	0,02	0,01	0,10	0,06
	Verrucomicrobiae	*Chthoniobacterales*	0,62	0,97	0,18	0,00	0,00	0,00	0,02	0,10	0,07
		Opitutales	0,01	0,00	0,00	0,00	0,00	0,00	0,00	0,02	0,00
		Pedosphaerales	0,01	0,01	0,00	0,00	0,00	0,00	0,00	0,01	0,03
		unclassified S-BQ2-57 soil group	0,00	0,00	0,00	0,00	0,00	0,00	0,00	0,00	0,00
		unclassified Verrucomicrobiae	0,00	0,00	0,00	0,00	0,00	0,00	0,01	0,02	0,02
		Verrucomicrobiales	0,22	1,01	0,16	0,00	0,02	0,04	0,16	0,15	0,14
WPS-2	*Unclassified WPS-2*	*Unclassified WPS-2*	0,00	0,07	0,02	0,00	0,00	0,00	0,00	0,02	0,01
Unknown Bacteria	*Unknown Bacteria*	*Unknown Bacteria*	0,00	0,03	0,00	0,00	0,00	0,00	0,03	0,01	0,01

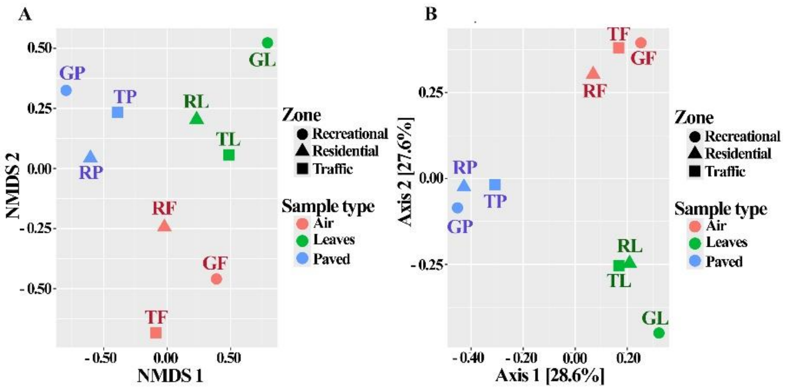

Figure S2. Non-metric multidimensional scaling (NMDS) (A) and principal coordinate analysis (PCoA) (B) of bacterial communities

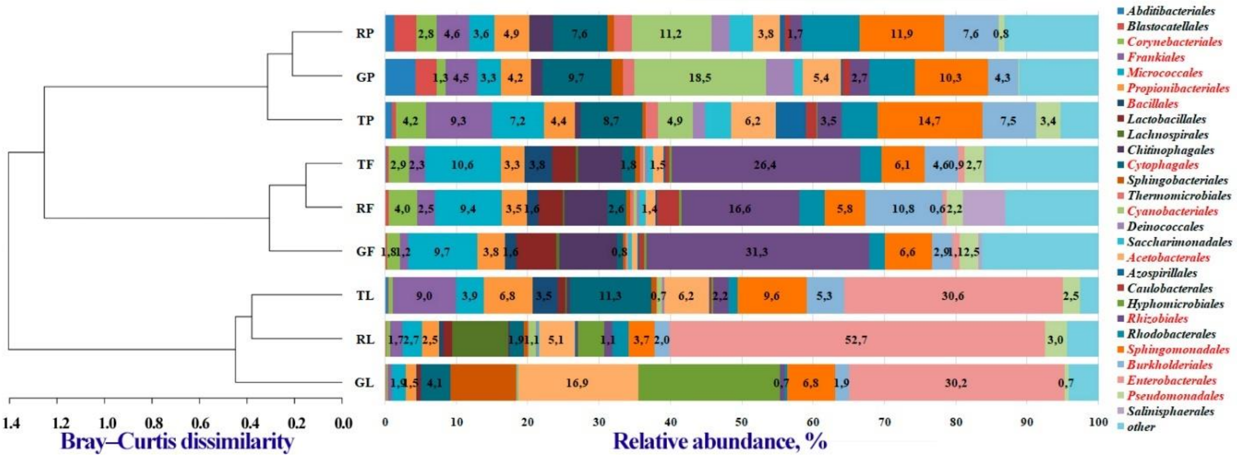

Figure S3. Distribution of the most abundant bacterial classes (relative abundance > 0.1%) identified in functional zones and biotopes of Moscow. Classes with relative abundance ≤0.1% were considered as "other". The clustering analysis of the class-level communities based on the calculation of the pairwise Bray-Curtis dissimilarity matrix is also reported. The first letter of the abbreviation refers to the functional zone: T-traffic, R-residential, G-recreational; and the last letter refers to the biotope: L-leaves, P-paved surface, F-filter/PM10 air.

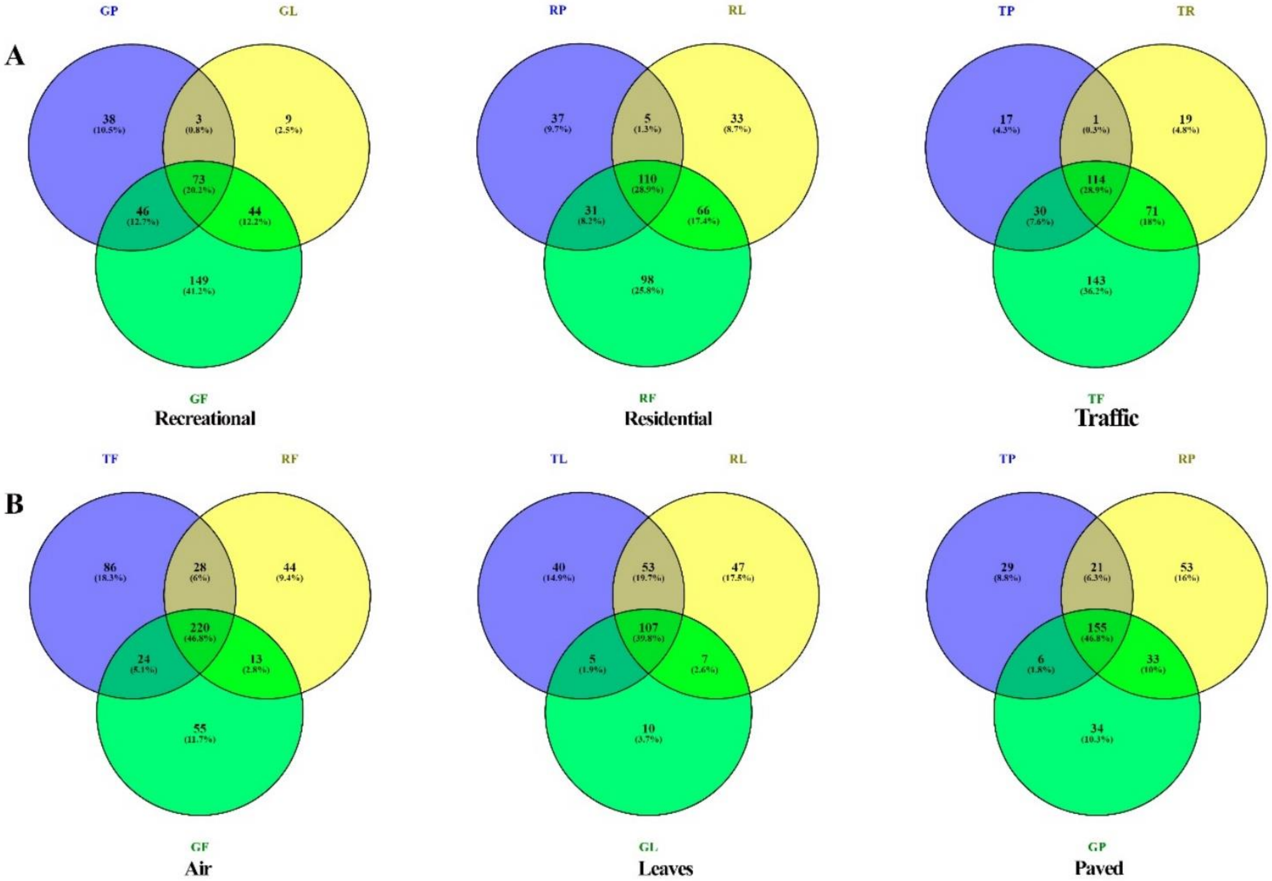

Figure S4. Venn diagrams illustrating the number of unique and shared bacterial ASVs at the level of genus among dust particles from Moscow that were detected in three urban zones.

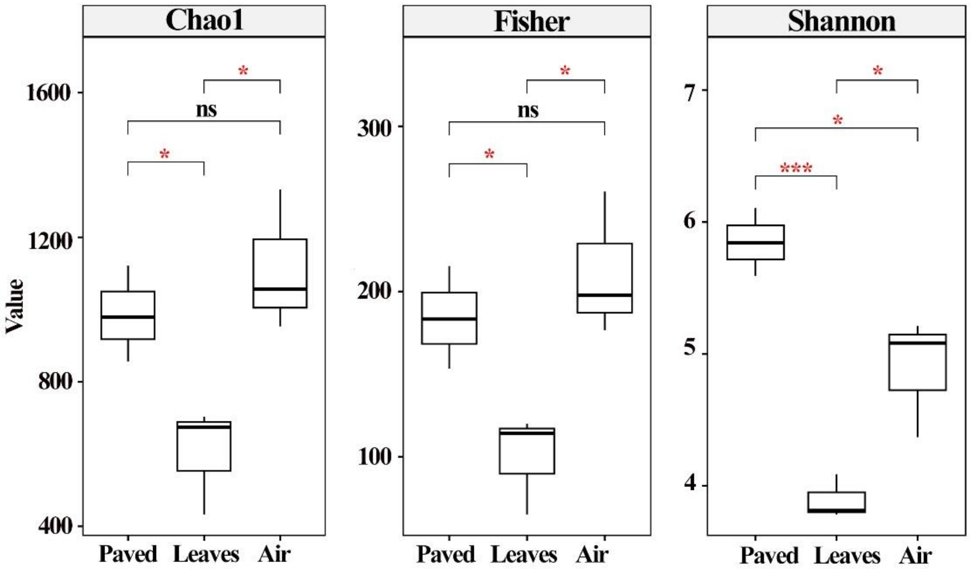

Figure S5. Comparisons of Chao1 richness, Fisher and Shannon diversity of bacterial communities between Moscow biotopes. Horizontal lines indicate significance levels between groups (* $p < 0.05$, *** $p < 0.001$).

Table S3. Total abundances of genes included in the respective KEGG modules ($p < 0.05$)

Module	Air	Leaves	Paved	Order
**Carbapenem resistance. [MD:M00851] [PATH:map01501]**	1409.99	101.75	165.25	Air > Paved > Leaves
**Cationic antimicrobial peptide (CAMP) resistance. VraFG transporter [MD:M00730]**	4472.50	541.50	16.00	Air > Leaves > Paved
**Cationic antimicrobial peptide (CAMP) resistance. dltABCD operon [MD:M00725]**	26154.51	8733.38	436.20	Air > Leaves > Paved

**Cationic antimicrobial peptide (CAMP) resistance. lysyl-phosphatidylglycerol (L-PG) synthase MprF [MD:M00726]**	53154.40	17790.64	21423.24	Air > Paved > Leaves
**Cationic antimicrobial peptide (CAMP) resistance. protease PgtE [MD:M00744]**	12082.12	59774.14	2140.56	Leaves > Air > Paved
**Imipenem resistance. repression of porin OprD [MD:M00745]**	42263.50	50522.59	9408.65	Leaves > Air > Paved
**Multidrug resistance. efflux pump AbcA [MD:M00700]**	7845.14	4998.45	338.55	Air > Leaves > Paved
**Multidrug resistance. efflux pump AcrEF-TolC [MD:M00696]**	275.68	25748.31	0.00	Leaves > Air > Paved
**Multidrug resistance. efflux pump AdeABC [MD:M00649] [PATH:map01501]**	2146.18	2831.68	3253.69	Paved > Leaves > Air
**Multidrug resistance. efflux pump BpeEF-OprC [MD:M00698]**	90791.60	19136.97	41314.73	Air > Paved > Leaves
**Multidrug resistance. efflux pump MdtEF-TolC [MD:M00697]**	6244.35	126163.11	109.78	Leaves > Air > Paved
**Multidrug resistance. efflux pump MepA [MD:M00705]**	961.99	295.00	905.65	Air > Paved > Leaves
**Multidrug resistance. efflux pump MexAB-OprM [MD:M00718]**	144728.50	131084.40	161897.93	Paved > Air > Leaves
**Multidrug resistance. efflux pump MexEF-OprN [MD:M00641]**	16775.21	9439.94	3595.01	Air > Leaves > Paved
**Multidrug resistance. efflux pump MexPQ-OpmE [MD:M00769]**	1026.25	482.25	24.00	Air > Leaves > Paved
**Multidrug resistance. efflux pump MexXY-OprM [MD:M00643]**	317.32	16.00	2.00	Air > Leaves > Paved
**Multidrug resistance. efflux pump NorB [MD:M00702]**	5123.00	3934.89	21.00	Air > Leaves > Paved
**Multidrug resistance. efflux pump QacA [MD:M00714]**	383.00	30.00	4.00	Air > Leaves > Paved
**Multidrug resistance. repression of porin OmpF [MD:M00746]**	560.48	69784.23	5.84	Leaves > Air > Paved
**Tetracycline resistance. efflux pump Tet38 [MD:M00704]**	2990.56	1918.39	189.23	Air > Leaves > Paved
**Vancomycin resistance. D-Ala-D-Lac type [MD:M00651]**	31846.46	53109.72	63422.08	Paved > Leaves > Air
**Vancomycin resistance. D-Ala-D-Ser type [MD:M00652]**	2048.00	3798.98	27.00	Leaves > Air > Paved
**beta-Lactam resistance. Bla system [MD:M00627]**	82031.57	50198.80	103889.25	Paved > Air > Leaves

Figure S6. Numbers of heterotrophs in the bacterial communities of air microparticles, leaf and paved dust

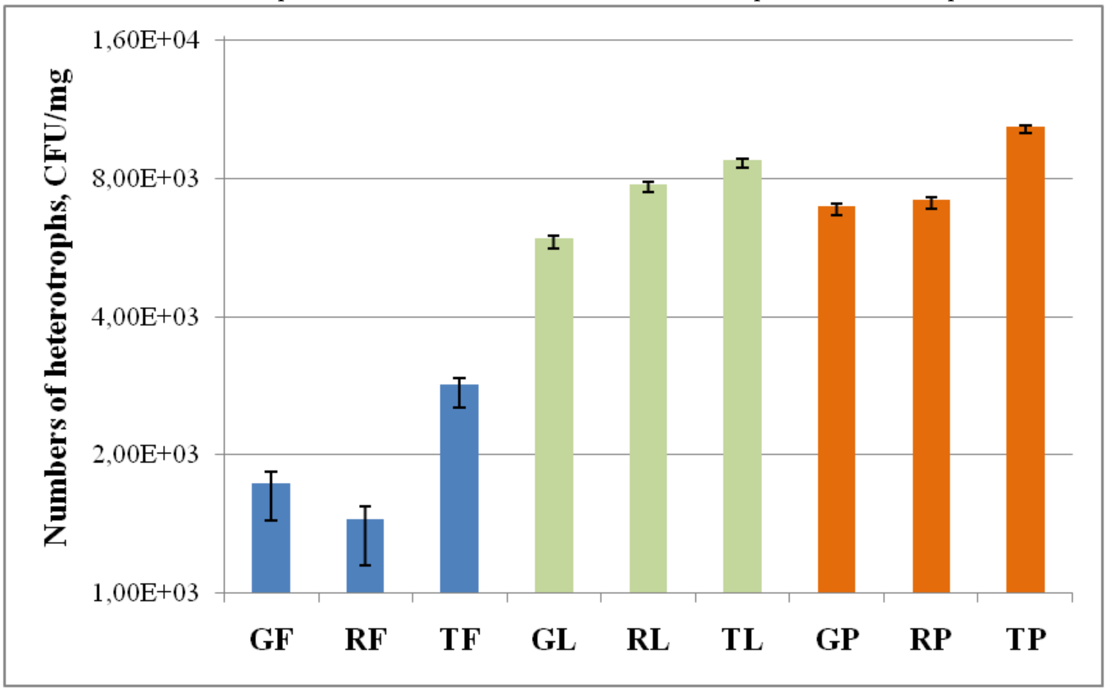

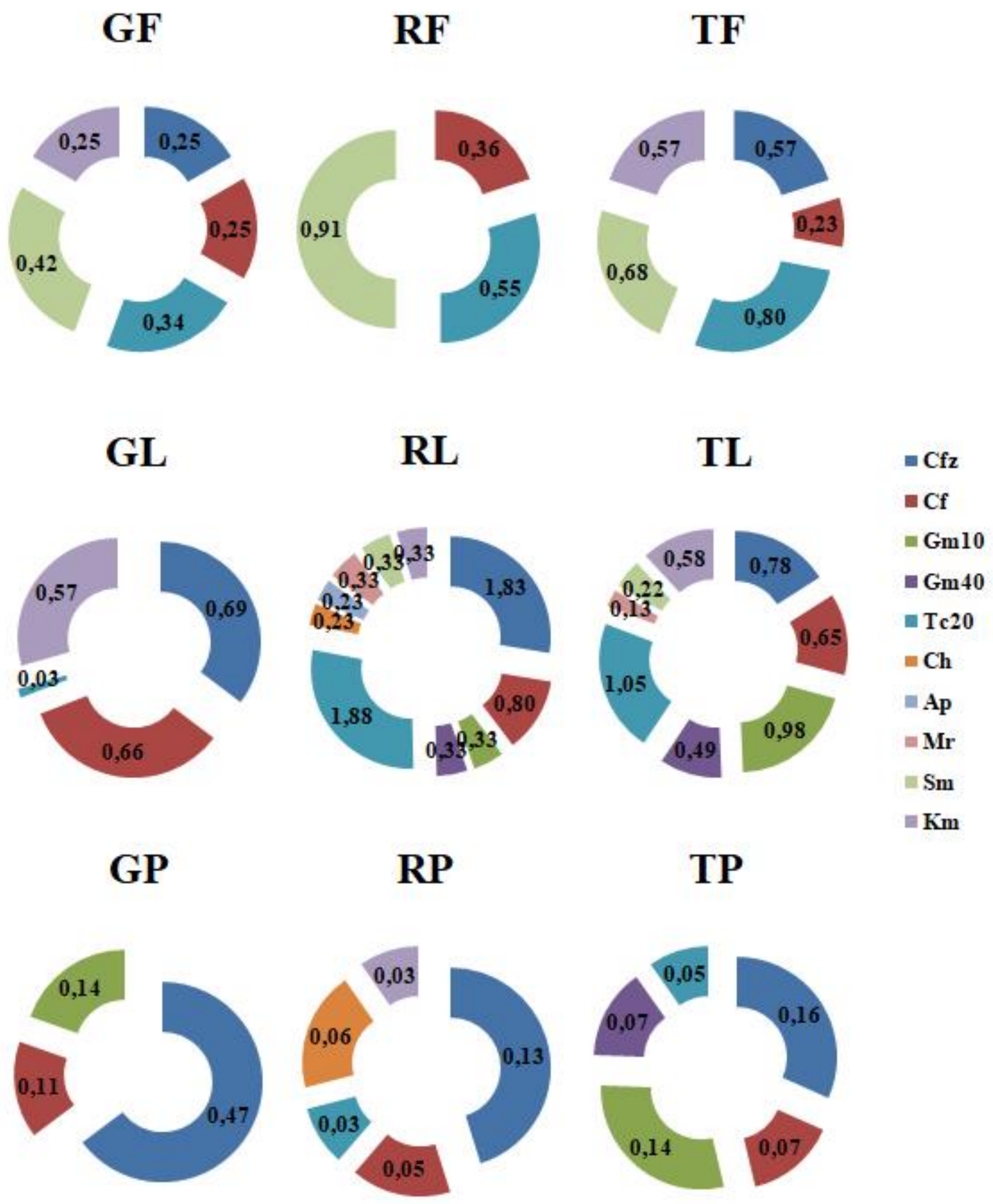

Figure S7. Total number of antibiotic-resistant bacteria grown on individual antibiotics from total heterotrophic bacteria. Data are shown as percentages.
Antibiotics: Meropenem (Mr), Ceftazidime (Cfz), Cefepime (Cf), Tetracycline (Tc), Chloramphenicol (Ch), Amikacin (Ak), Kanamycin (Km), Streptomycin (Sm) and Gentamicin (Gm10 μg/ml and Gm 40 μg/ml).

Table S4. Representation of genera of culturable antibiotic-resistant strains in the bacterial communities of selected dust particles

**GP**	*Pseudarthrobacter*					
**RP**	*Pseudarthrobacter*	*Bacillus*	*Arthrobacter*			
**TP**	*Pseudarthrobacter*	*Bacillus*	*Brachybacterium*	*Microbacterium*		
**GL**	*Pseudarthrobacter*	*Bacillus*	*Rothia*			
**RL**	*Pseudarthrobacter*	*Bacillus*	*Chryseobacterium*	*Microvirga*	*Methylorubrum*	
**TL**	*Pseudarthrobacter*	*Bacillus*	*Acinetobacter*	*Microvirga*	*Brachybacterium*	*Rothia*
**GF**	*Brevibacterium*	*Chryseomicrobium*	*Acinetobacter*	*Rahnella*	*Micrococcus*	
**RF**	*Brevibacterium*	*Chryseomicrobium*	*Chryseobacterium*	*Rahnella*		
**TF**	*Brevibacterium*	*Chryseomicrobium*	*Acinetobacter*	*Rahnella*	*Brachybacterium*	

Table S5. Relative abundance of genera of culturable antibiotic-resistant strains in the bacterial communities of selected dust particles

	GP	RP	TP	GL	RL	TL	GF	RF	TF
Acinetobacter		0.03	0.02	0.03	0.95	0.06	1.23	0.91	0.93
Arthrobacter	1.22	1.50	2.69	0.16	0.73	1.03	0.76	1.97	2.18
Bacillus	0.01	0.01	0.02	0.17	0.25	3.34	0.93	0.90	1.05
Brachybacterium			0.02			0.01	0.09	0.06	0.16
Brevibacterium							0.06	0.10	0.05
Chryseobacterium	0.82	0.66	0.17	0.02	0.07	0.05	0.48	0.76	0.63
Chryseomicrobium						0.02	0.04	0.04	0.20
Methylorubrum	1.37	0.68	2.20	0.65	0.71	1.91	0.46	0.73	0.65
Microbacterium	0.04	0.04	0.10	0.02	0.11	0.03	0.27	0.42	0.36
Micrococcus				0.03	0.13	0.09	2.01	1.31	1.03
Microvirga		0.09			0.01	0.02		0.02	0.06
Pseudarthrobacter	0.24	0.14	0.17	0.09	0.15	0.50	0.51	0.49	0.43
Rahnella							0.02	0.02	0.05
Rothia				0.07	0.31	0.19	1.64	1.56	0.81

Table S6. Cytoscape data for network visualisation showing the interaction between different bacterial ASVs at the species level in dust samples.

Name	Node.degree	No. module	Zi	Pi
Abditibacterium sp.	2	8	0	0
*Acidiphilium sp. 3**	1	2	-1.72622274380808	0
*Acidiphilium sp. 4**	1	5	-0.589506344746563	0
Acinetobacter lwoffii	13	2	1.11369209277941	0
Alkanindiges sp. 1	2	9	0	0
Amaricoccus sp. 1	5	0	-0.172870199746124	0
Amaricoccus sp. 2	7	2	-0.306265325514338	0
Arthrobacter agilis	1	12	0	0
Arthrobacter sp. 1	1	9	0	0
Arthrobacter sp. 2	4	2	-1.01624403466121	0
Arthrobacter sp. 3	3	2	-1.25290360437684	0
Blastocatella sp. 1	9	0	0.727874525246839	0.197530864197531
Blastocatella sp. 4	13	2	1.11369209277941	0
Blastocatella sp. 5	11	2	0.64037295334816	0
Blastocatella sp. 9	1	5	-0.589506344746563	0
Blastococcus aggregatus	2	1	-0.768657724359598	0
Blastococcus sp. 1	8	1	1.65868245782861	0.21875
Blastococcus sp. 3	7	1	1.65868245782861	0
Blastococcus sp. 4	2	7	0	0
Brevundimonas sp. 2	2	1	-0.768657724359598	0
Brevundimonas sp. 3	5	0	-0.172870199746124	0
Cellulomonas sp. 2	1	3	-1.17260393995586	0
Chryseobacterium sp. 3	2	7	0	0
Clostridium bowmanii	8	1	0.202278348515684	0.59375
Clostridium sensu stricto 1 sp. 1	1	11	0	0
Deinococcus aerolatus	13	0	2.22911573356844	0
Deinococcus aquatilis	2	5	-0.168430384213304	0
Deinococcus sp. 1	3	6	-0.267261241912425	0

Deinococcus sp. 2	2	1	-0.768657724359598	0
Exiguobacterium sp. 1	4	2	-1.01624403466121	0
Friedmanniella sagamiharensis	8	0	-0.172870199746124	0.5625
Friedmanniella sp. 1	8	0	0.427626283582518	0.21875
Friedmanniella sp. 3	5	1	0.687746384953325	0
Friedmanniella sp. 4	1	8	0	0
Friedmanniella sp. 5	5	0	-0.773366683074767	0.48
Galbitalea sp. 1	11	2	0.64037295334816	0
Geminicoccus sp.	5	6	1.33630620956212	0
Hymenobacter actinosclerus	2	1	-0.768657724359598	0
Hymenobacter elongatus	1	15	0	0
Hymenobacter sp. 10	6	0	0.127378041918197	0
Hymenobacter sp. 29	1	0	-1.37386316640341	0
Hymenobacter sp. 3	5	1	0.202278348515684	0.32
Hymenobacter sp. 33	4	0	-0.473118441410446	0
Hymenobacter sp. 50	1	14	0	0
Hymenobacter sp. 6	5	1	0.202278348515684	0.32
Hymenobacter sp. 7	5	6	1.33630620956212	0
Klenkia terrae	10	0	1.32837100857548	0
Knoellia sp. 1	3	3	1.17260393995586	0
Kocuria rosea	19	2	2.53364951107316	0
Marisediminicola sp. 1	4	2	-1.01624403466121	0
Marmoricola sp. 1	7	1	-0.283189687921957	0.489795918367347
Massilia sp. 1	1	5	-0.589506344746563	0
Massilia sp. 10	6	2	-0.542924895229962	0
Massilia sp. 15	1	1	-1.25412576079724	0
Massilia sp. 7	1	13	0	0
Massilia violaceinigra	3	4	0	0.444444444444444
Methylobacterium-Methylorubrum sp. 1	11	2	-0.306265325514338	0.462809917355372
Methylobacterium-Methylorubrum sp. 10	3	4	1.1180339887499	0
Methylobacterium-Methylorubrum sp. 2	10	0	1.32837100857548	0
Methylobacterium-Methylorubrum sp. 3	14	2	1.35035166249503	0
Methylobacterium-Methylorubrum sp. 4	9	0	1.02812276691116	0
Methylobacterium-Methylorubrum sp. 5	9	2	-0.0696057557987133	0.197530864197531
Methylobacterium-Methylorubrum sp. 6	12	2	0.877032523063785	0
Methylobacterium-Methylorubrum sp. 7	4	0	-0.473118441410446	0
Modestobacter sp. 1	4	0	-0.473118441410446	0
Mycobacterium sp. 1	8	1	1.65868245782861	0.21875
Mycolicibacterium doricum	9	5	2.77910133951951	0
Nakamurella flavida	16	0	2.52936397523277	0.2265625
Nakamurella sp. 1	6	1	1.17321442139097	0
Nakamurella sp. 2	11	2	0.64037295334816	0
Nakamurella sp. 3	4	6	0.534522483824849	0
Nocardioides alpinus	4	4	-1.1180339887499	0.625
Nocardioides sp. 1	4	0	-0.473118441410446	0
Nocardioides sp. 2	1	10	0	0
Nocardioides sp. 3	6	0	0.127378041918197	0
Nocardioides sp. 4	3	3	1.17260393995586	0
Noviherbaspirillum sp. 1	4	0	-0.473118441410446	0
Noviherbaspirillum sp. 2	11	0	1.6286192502398	0

Noviherbaspirillum sp. 3	1	0	-1.37386316640341	0
Noviherbaspirillum sp. 4	11	2	0.64037295334816	0
Novosphingobium sp. 1	1	6	-1.87082869338697	0
Novosphingobium sp. 8	4	6	0.534522483824849	0
Ornithinibacter sp. 1	4	0	-0.473118441410446	0
Ornithinimicrobium sp. 1	2	7	0	0
Paracoccus contaminans	5	0	-0.172870199746124	0
Paracoccus sp. 1	6	2	-0.542924895229962	0
Paracoccus sp. 2	6	2	-0.779584464945587	0.277777777777778
Paracoccus sp. 3	1	0	-1.37386316640341	0
Paracoccus sp. 4	1	8	0	0
Pedobacter sp. 7	9	2	0.167053813916911	0
Polaromonas sp. 1	1	3	-1.17260393995586	0
Polaromonas sp. 2	7	2	-0.306265325514338	0
Polaromonas sp. 3	2	0	-1.07361492473909	0
Prevotella_7 histicola	16	2	1.82367080192628	0
Pseudarthrobacter sp. 1	2	3	-1.17260393995586	0.5
Pseudarthrobacter sp. 2	1	1	-1.25412576079724	0
Psychrobacter sp. 1	1	10	0	0
Psychroglaciecola sp. 2	2	1	-0.768657724359598	0
Qipengyuania sp. 2	2	1	-1.25412576079724	0.5
Rhodococcus sp. 1	1	9	0	0
Romboutsia sp. 1	2	3	0	0
Roseomonas aquatica	2	0	-1.07361492473909	0
Rubellimicrobium sp. 1	2	10	0	0
Rubellimicrobium sp. 11	3	6	-0.267261241912425	0
Rubellimicrobium sp. 2	4	5	0.673721536853215	0
Rubellimicrobium sp. 3	15	2	1.58701123221066	0
Rubellimicrobium sp. 4	10	2	0.403713383632536	0
Rubellimicrobium sp. 5	4	2	-1.01624403466121	0
Rubellimicrobium sp. 8	9	0	1.02812276691116	0
Skermanella aerolata	3	3	1.17260393995586	0
Sphingobium sp. 1	2	1	-0.768657724359598	0
Sphingomonas astaxanthinifaciens	6	1	1.17321442139097	0
Sphingomonas humi	7	1	1.65868245782861	0
Sphingomonas jaspsi	4	2	-1.01624403466121	0
Sphingomonas sp. 1	9	2	-0.542924895229962	0.493827160493827
Sphingomonas sp. 10	2	3	0	0
Sphingomonas sp. 11	1	5	-0.589506344746563	0
Sphingomonas sp. 13	4	1	0.202278348515684	0
Sphingomonas sp. 14	3	4	1.1180339887499	0
Sphingomonas sp. 17	2	2	-1.48956317409246	0
Sphingomonas sp. 3	9	2	0.167053813916911	0
Sphingomonas sp. 30	2	3	0	0
Sphingomonas sp. 4	7	0	0.427626283582518	0
Sphingomonas sp. 6	7	2	-0.542924895229962	0.244897959183674
Sphingomonas sp. 7	2	1	-0.768657724359598	0
Sphingomonas sp. 8	1	0	-1.37386316640341	0
Sphingorhabdus rigui	1	3	-1.17260393995586	0
Spirosoma sp. 10	1	5	-0.589506344746563	0

Spirosoma sp. 3	2	5	-0.168430384213304	0
unclassified Acetobacteraceae 3	1	14	0	0
unclassified Bacillaceae 1	1	11	0	0
unclassified Blastocatellaceae 4	2	5	-0.168430384213304	0
unclassified Frankiales 1	6	0	0.127378041918197	0
unclassified Frankiales 2	1	13	0	0
unclassified Frankiales 3	3	3	1.17260393995586	0
unclassified Frankiales 4	9	2	0.167053813916911	0
unclassified Intrasporangiaceae 2	1	15	0	0
unclassified Micrococcales 1	2	1	-0.768657724359598	0
unclassified Microtrichales 1	1	4	-1.1180339887499	0
unclassified Oxalobacteraceae 1	3	6	-0.267261241912425	0
unclassified Thermomicrobiales 6	4	0	-0.473118441410446	0
unclassified Thermomicrobiales 7	4	0	-0.473118441410446	0
unclassified Thermomicrobiales 8	5	0	-0.172870199746124	0
Variovorax paradoxus	10	2	0.403713383632536	0
Variovorax sp. 1	5	2	-0.779584464945587	0
Williamsia sp. 1	3	1	-0.283189687921957	0
Williamsia sp. 2	3	6	-1.0690449676497	0.444444444444444
Williamsia sp. 4	1	12	0	0

Red font - opportunistic microorganism; green font - microorganisms found in bacteraemia and inflammatory processes; * - bacteria with the same names but different nucleotide sequences are indicated; red circle - intramodule hubs, i.e. had the maximum number of neighbours within their modules; green circle - connectors for communication with other modules.
